# Kasumi leukemia cell lines: characterization of tumor genomes with ethnic origin and scales of genomic alterations

**DOI:** 10.1007/s13577-020-00347-5

**Published:** 2020-03-16

**Authors:** Fumio Kasai, Hiroya Asou, Midori Ozawa, Kazuhiko Kobayashi, Hiroyuki Kuramitsu, Motonobu Satoh, Arihiro Kohara, Yasuhiko Kaneko, Machiko Kawamura

**Affiliations:** 1 Japanese Collection of Research Bioresources (JCRB) Cell Bank, National Institutes of Biomedical Innovation, Health and Nutrition, Saito-Asagi 7-6-8, Ibaraki, Osaka 567-0085 Japan; 2 Medicine Development Unit, Eli Lilly, Kobe, 651-0086 Japan; 3 Department of Clinical Laboratory, Saitama Cancer Center, Saitama, 362-0806 Japan; 4 Research Institute for Clinical Oncology, Saitama Cancer Center, Saitama, 362-0806 Japan; 5 Department of Hematology, Saitama Cancer Center, Saitama, 362-0806 Japan

**Keywords:** Chromosome instability, Fusion gene, Tumor genome size, Ancestry information, *TP53* alteration

## Abstract

Kasumi-1 has played an important role in an experimental model with t(8;21) translocation, which is a representative example of leukemia cell lines. However, previous studies using Kasumi-1 show discrepancies in the genome profile. The wide use of leukemia cell lines is limited to lines that are well-characterized. The use of additional cell lines extends research to various types of leukemia, and to further explore leukemia pathogenesis, which can be achieved by uncovering the fundamental features of each cell line with accurate data. In this study, ten Kasumi cell lines established in Japan, including five that were previously unknown, have been characterized by SNP microarray and targeted sequencing. SNP genotyping suggested that the genetic ancestry in four of the ten Kasumi cell lines was not classified as Japanese but covered several different east-Asian ethnicities, suggesting that patients in Japan are genetically diverse. *TP53* mutations were detected in two cell lines with complex array profiles, indicating chromosomal instability (CIN). A quantitative assessment of tumor genomes at the chromosomal level was newly introduced to reveal total DNA sizes and Scales of Genomic Alterations (SGA) for each cell line. Kasumi-1 and 6 derived from relapsed phases demonstrated high levels of SGA, implying that the level of SGA would reflect on the tumor progression and could serve as an index of CIN. Our results extend the leukemia cellular resources with an additional five cell lines and provide reference genome data with ethnic identities for the ten Kasumi cell lines.

**Electronic supplementary material:**

The online version of this article (10.1007/s13577-020-00347-5) contains supplementary material, which is available to authorized users.

## Introduction

Leukemia genomes are primarily characterized by abnormal karyotypes, which often include the formation of a fusion gene [1]. Chromosomal translocations are responsible for leukemia initiation, but are usually not sufficient for further development of the condition [2]. Chromosome instability (CIN) is linked to tumor progression [3] and causes genomic diversity [4]. In addition, recurrent hotspot mutations have been identified in association with leukemogenesis [5]. Both large-scale changes at chromosome level and nucleotide changes at sequence level contribute to a diverse array of leukemia genomes, reflecting a broad range of subtypes [6, 7]. While sequence analysis provides data at the highest resolution, CIN is mostly based on qualitative data. Quantitative analysis of large-scale genomic alterations is required to assess CIN status and would improve the precise disease classification.

Kasumi cell lines have been established at Hiroshima University from patients with leukemia in Japan since 1989. They were named after the location of the laboratory in the Kasumi area of Hiroshima city [8] (Table 1). The aim for establishing these cell lines was to provide a useful research model harboring leukemia-specific chromosomal/gene abnormalities. A fusion gene, *RUNX1-RUNX1T1*, is well recognized as an important diagnostic and prognostic marker frequently observed in acute myeloid leukemia, according to the French–American–British classification M2 subtype (AML-M2), which was first identified in the Kasumi-1 cell line [9, 10]. Kasumi-1, 2, 3, 4 and 6 had been deposited in three major cell banks, ATCC, DSMZ and JCRB, directly from a laboratory which established the lines. An additional five cell lines have been listed only in the JCRB cell bank (Table 1). Although two comprehensive databases of cancer cell lines, COSMIC and CCLE, are available from the Sanger Institute and the Broad Institute, respectively, information about leukemia cell lines is limited to certain commonly-used cell lines. Kasumi-1 appears in both COSMIC and CCLE, and the latter also includes Kasumi-2 and 6. However, other Kasumi cell lines have not been fully exploited because their cells are poorly characterized.

**Table 1:** A series of Kasumi leukemia cell lines

Name	Type	Age	Sex	JCRB	DSMZ	ATCC	Features [selected references]
Kasumi-1	AML-M2	7	M	JCRB1003	ACC 220	CRL-2724	First AML cell line with t(8;21) [8], Primary culture from 2nd relapsed BM after BMT
Kasumi-2	BCP-ALL	15	M	JCRB1395	ACC 526		Non-productive BCR [11]
Kasumi-3	AML-M0	57	M	JCRB1004	ACC 714	CRL-2725	*EVI1* activation associated with t(3;7)(q27;q22) [12]
Kasumi-4	CML-BC	6	F	JCRB0161		CRL-2726	t(9;22;11)(q34;q11;q13) with *BCR-ABL* fusion, inv(3)(q21q26) with *EVI1* overexpression [13]
Kasumi-5	T-ALL	24	M	JCRB1398			Sensitivity to a RhoA kinase inhibitor, Y27632
Kasumi-6	AML-M2	64	M	JCRB1024	ACC 686	CRL-2775	Dominant-negative mutation in the *C/EBP alpha* gene [14], Established from PB when relapsed
Kasumi-7	BCP-ALL	29	F	JCRB1401			
Kasumi-8	BCP-ALL	48	M	JCRB1403			
Kasumi-9	BCP-ALL	19	M	JCRB1409			
Kasumi-10	BCP-ALL	6 M	F	JCRB1410			

Cell lines have attractive features, namely their ‘continuous’ or ‘immortalized’ abilities. This enables us to use the same cellular material across different laboratories and allows us to compare results using cell lines. However, three previous studies which analyzed Kasumi-1 by DNA microarray showed discordance in the genome profiles [15–17] (Table S1). Analysis of MCF7 strains revealed genetic evolution of cancer cell lines during cell culture [18]. This implies that tumor genomes change during in vitro cell culture, which can be explained by an in vitro clonal evolution model [19]. Analysis of cell lines obtained from a public registry provides results which ensure reproducibility, leading to an accurate reference.

A panel of 100 leukemia and lymphoma cell lines, LL-100, has been reported [20]; however, most of them, 80 of the 100 cell lines, are included in the CCLE and/or COSMIC databases. In addition, all the cell lines have already appeared in publications and no novel cell lines are introduced in the panel. Because the panel does not adequately cover various types of leukemia, additional cell lines are required for further investigation of the underlying molecular mechanisms in leukemogenesis. We performed SNP array and sequence analyses in ten Kasumi cell lines to obtain their genome reference data. Changes at chromosome level in tumor genomes were assessed by measurements of gains, losses and uniparental disomy (UPD), shown as Scales of Genomic Alterations (SGA). Amplicon sequencing detected pathogenic mutations and candidate mutations with their allele frequencies. An RNA sequencing panel identified fusion genes in five cell lines, with accompanying expression levels. Our study demonstrates quantitative assessment of leukemia genomes and adds ethnic information on each cell line.

## Materials and methods

### Cell lines, cell culture and DNA extraction

Kasumi-1–10 cell lines have been registered with the JCRB cell bank (Table 1) and are available for distribution upon request. When the cells were defrosted, the cells were cultured at a higher concentration, and the culture disk or flask was slanted to achieve a high cell density at the lower end before cells became stable. In addition, increasing the heat-inactivated FBS concentration to 20% in the medium could help to promote growth after defrosting. Because of a lot-to-lot variation in FBS, it has been carefully selected by evaluating a range of different lots (Table S2). It is noted that GM-CSF is required for cultivation of Kasumi-4 and 6. Two normal fibroblast cell lines, SF-TY (JCRB0075) and TIG-7–20 (JCRB0511), were used as controls during sequence variant analysis. Culture conditions and data sets from the standard quality control for each cell line are available in the JCRB Cell Bank website at https://cellbank.nibiohn.go.jp/english. Genomic DNA was extracted from cultured cells using the AllPrep DNA/RNA Mini Kit (Qiagen 80204).

### SNP microarray

DNA copy number and genotyping were examined by microarray using a high density chip, CytoScan HD array (Thermo Fisher Scientific). The data analysis was performed based on the GRCh37 (hg19) reference using the Chromosome Analysis Suite software, ChAS 4.0 (Thermo Fisher Scientific). To measure genomic changes at chromosomal level, regions of gains, losses and uniparental disomy (UPD) larger than 1 Mb were counted. Compared with normal diploid male and female DNA sizes of 5977 Mb and 6073 Mb respectively, from the human genome reference hg19, nuclear DNA size of each cell line was estimated from the difference between gains and losses. The sum of sizes from gains, losses and UPD were calculated as the Scale of Genomic Alterations (SGA).

### Amplicon sequencing

Mutation analysis by target sequencing was conducted using a multiplex panel, Oncomine™ Myeloid Research Assay (Thermo Fisher Scientific, A36941), consisting of 40 DNA genes analyzed by 526 amplicons and 29 RNA genes representing 700 fusion isoforms found in major myeloid disorders. An On-Demand panel, IAD178152, consisting of 6236 amplicons was designed and applied to AML cell lines and the two normal cell lines. This custom panel covered 286 leukemia-related genes and extended to 1.19 Mb in total (Table S2). Ethnic origin of each cell line was assessed by the Precision ID Ancestry Panel (Thermo Fisher Scientific, A25642). Sequence libraries and templates were prepared using the Ion AmpliSeq Kit for Chef DL8 (Thermo Fisher, A29024) and the Ion PGM Hi-Q View Chef Kit (Thermo Fisher Scientific, A29902), respectively. Sequencing was run on the Ion PGM using the Ion 318 Chip v2 BC (Thermo Fisher Scientific, 4488150). Reads were aligned to the hg19 reference and the analysis was carried out using the Ion Torrent Suite and the Ion Reporter (Thermo Fisher Scientific). To eliminate SNPs from variants, variants specific for each Kasumi cell line were extracted by comparisons with the two normal cell lines. Filter settings used for variant analysis of sequence data from the custom panel are given in Table S3. To obtain biogeographic ancestry information, sequence data from the ID panel were analyzed using the Ion Torrent HID SNP Genotyper Plugin (Thermo Fisher Scientific).

### Flow cytometric immunophenotyping

Cells were washed and incubated for 20 min at 4 °C with antibodies and their isotype controls are listed in Table S4. The cells were washed with 5% FBS/PBS twice and resuspended in 4% PFA, and analyzed on a flow cytometer, FACSCanto using the FlowJo software (BD Biosciences).

## Results

### Whole genome profiles

SNP microarray profiles of each cell line are shown in Fig. S1 and described in Table S5. Microarray data, CHP and CEL files, have been submitted to the NCBI repository databases under the BioProject ID PRJNA598005. Copy number alterations were identified in nine of the ten cell lines, with the exception of Kasumi-4. Although the array profile of Kasumi-4 represented an apparently normal female, a translocation, t(9;11;22), which forms the *BCR-ABL1* fusion has been reported in Kasumi-4. Kasumi-10 has been established from a female infant with B-cell precursor acute lymphoblastic leukemia (BCP-ALL), which does not show detectable copy number changes in autosomes, but has a balanced translocation, t(11;19)(q23;p13.3). Due to a limitation of this technique [21], array profiles show DNA gross changes, but miss balanced translocations. There was in discordance with the sex chromosomes of Kasumi-10 which showed XXY, which could be explained by disorders of sexual development [22]. Kasumi-1 from a male patient had one copy of X chromosome without a corresponding Y chromosome, consistent with the clinical data, indicating that the loss occurred before cell culture.

**Fig. 1:**
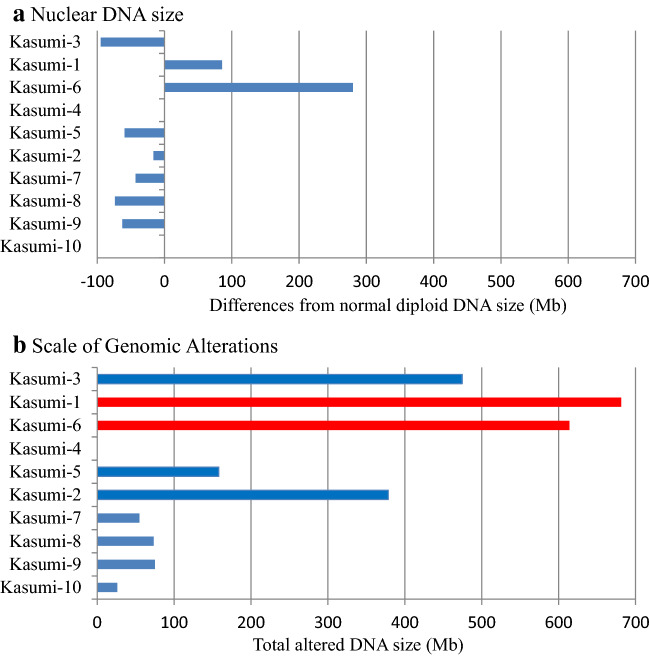
Assessment of tumor genomes. **a** Gross changes of nuclear DNA in each Kasumi cell line are calculated from SNP microarray profiles. Kasumi-4 and 10 have no gross changes. Kasumi-1 and 6 increase the DNA size and others reduce within 100 Mb. **b** Overall genomic changes are shown by Scale of Genomic Alterations. Although the changes in DNA amounts are apparently small, cell lines with *TP53* mutations indicated in red demonstrate high SGA

Gains and losses were observed in 6 and 8 cell lines, respectively (Table 2, S6). Trisomy 10 was observed in Kasumi-1 and 6, demonstrating a characteristic feature of AML-M2 [23]. Loss of heterozygosity (LOH) resulting in one copy of alleles did not occur in a whole chromosome but in partial regions. DNA size compared with normal diploid was calculated from 98.4 to 104.7% for the three AML cell lines and from 98.8 to 99.7% for the five BCP-ALL cell lines (Fig. 1a). The largest difference among the 10 cell lines was found in Kasumi-6, which increased its DNA size to 280.2 Mb. SNP profiles revealed that UPD, equivalent to copy neutral LOH consisting of homozygous DNA copies, occurred in 7 cell lines, shown as ‘hmz’ in Table S5. Each case was detected in only parts of chromosomes, which were distinct between samples. The scale of regions involving DNA changes calculated as Scale of Genomic Alterations (SGA) showed that three AML cell lines (Kasumi-1, 3, and 6) had larger sizes of altered regions than ALL cell lines.

**Table 2:** Quantitative assessment of large-scale genomic changes

		Gain			Loss			DNA size		UPD			SGA			NCBI GEO
		Regions	(Mb)	(%)	Regions	(Mb)	(%)	(Mb)	(%)	Regions	(Mb)	(%)	Regions	(Mb)	(%)	Accession
Kasumi-3	AML-M0	2	70.9	1.2	10	165.6	2.8	− 94.7	− 1.6	12	238.1	4.0	24	474.6	7.9	GSM4254131
Kasumi-1	AML-M2	11	348.5	5.8	11	262.9	4.4	85.6	+ 1.4	1	70.0	1.2	23	681.4	11.4	GSM4254129
Kasumi-6	AML-M2	6	336.4	5.6	2	56.2	0.9	280.2	+ 4.7	7	221.4	3.7	15	614.0	10.3	GSM4254134
Kasumi-4	CML-BC	0	0	0	0	0	0	0	0	0	0	0	0	0	0	GSM4254132
Kasumi-5	T-ALL	1	25.2	0.4	3	84.5	1.4	− 59.3	− 1.0	1	48.3	0.8	5	158.0	2.6	GSM4254133
Kasumi-2	BCP-ALL	2	181.1	3.0	10	197.4	3.3	− 16.3	− 0.3	0	0	0	12	378.5	6.3	GSM4254130
Kasumi-7	BCP-ALL	1	1.2	0.0	9	44.1	0.7	− 42.9	− 0.7	1	9.6	0.2	11	54.9	0.9	GSM4254135
Kasumi-8	BCP-ALL	0	0	0	5	73.6	1.2	− 73.6	− 1.2	0	0	0	5	73.6	1.2	GSM4254136
Kasumi-9	BCP-ALL	0	0	0	6	62.5	1.0	− 62.5	− 1.0	1	12.6	0.2	7	75.1	1.3	GSM4254137
Kasumi-10	BCP-ALL	0	0	0	0	0	0	0	0	1	26.2	0.4	1	26.2	0.4	GSM4254138

Normal total DNA sizes 5977 Mb for males (46,XY) and 6073 Mb for females(46,XX) based on the hg19 reference genome data

Because microarray analysis is performed using genomic DNA samples extracted from an admixture of cells, a non-integer copy number appears in the profiles when the cell population is heterogeneous. This was observed at 9q and 18q in Kasumi-9 (Figure S1), which showed copy numbers of 1.3 and indicated mosaic losses, reflecting heterogeneous cell populations.

Cryptic homozygous deletion measured between 300 and 1200 kb at the *CDKN2A/p16* locus (9p21.3) was observed in three ALL cell lines, which involved LOH across the boundary of the deletion (Figure S2A-C). Additional losses were detected in other 9p regions, which were different between the three samples. Kasumi-9 had two additional homozygous deletions at 9p23 and 9p21.3 which extended to 5 Mb and 1 Mb, respectively, implying the occurrence of complex rearrangements on its chromosome 9.

### Mutational signatures

Sequence variants detected by the Oncomine™ myeloid panel are listed in Table S7. Of them, hotspot mutations were identified in 7 cell lines (Table 3, S8). *TP53* mutations were detected in Kasumi-1 and 6, both of them exhibited 17p LOH in the array profiles (Figure S2D), resulting in a 100% allele frequency (Table S8). FMS-like tyrosine kinase 3 internal tandem duplication (*FLT3-ITD*) was detected in Kasumi-6 and -10, derived from elderly AML and infant ALL patients, respectively. Kasumi-1 has been used as a *KIT* mutant AML model [24], which was detected at 4q12 with 79.3% variant frequency under 4 copies, indicating duplications of the mutated allele. Additional analysis of six ALL cell lines were done using a custom panel provided variant lists (Table S9) and candidate pathogenic mutations (Table S10). Of them, variants detected in driver genes reported in ALL are shown in Table 3. A low frequency of variants can arise during cell culture. A missense mutation in *NSD1* was detected in all of the six cell lines at about a 1:3 frequency ratio, which would be explained by a nucleotide change after the gene duplication.

**Table 3:** Genomic features as a reference panel

	Type	Age	Sex	Population^a^	Fusion gene	Hotspot mutation	*TP53*	*FLT3*	Chromosome rearrangements	Array	*p16*	Candidate causative genes
Kasumi-3	AML-M0	57	M	JP		*ETV6*(12p13.2)			t(3;7)(q27;q22)	7p-, 12p13 LOH		
Kasumi-1	AML-M2	7	M	KR-HAN	*RUNX1-RUNX1T1*	*KIT*	p.Arg248Gln		-Y, t(8;21)(q21;q22)	+ 10, 17p-	LOH	
Kasumi-6	AML-M2	64	M	KR-JP		*WT1, STAG2,CEBPA*^b^	p.Tyr234Asp	ITD	add(12)(p11), add(13)(p11)	+ 10, 13q + , 17p-		
Kasumi-4	CML-BC	6	F	JP	*BCR-ABL1*	*GATA2*			t(9;22;11)(q34;q11;q13)	Normal		
Kasumi-5	T-ALL	24	M	HAN-TW	*NUP98-RAP1GDS1*^b^				t(4;11)(q21;p15)	3pUPD,12p-, 17p-		*KIT*, *NOTCH1*, *SETBP1*, *CREBBP*
Kasumi-2	BCP-ALL	15	M	JP	*TCF3-PBX1*	*HRAS*			t(1;19)(q23;p13)	1q + , 6q-,7p-, 7q +		*TAL1*, *TET2*, *KRAS*, *NF1*, *ATRX*
Kasumi-7	BCP-ALL	29	F	JP					t(4;9)(q21;?), del(9p)	del (*TMEM2*)	del	*ABL1*
Kasumi-8	BCP-ALL	48	M	JP	*BCR-ABL1*	*ETV6* (12p13.2)			t(9;22)(q34;q11)	12p13.2 LOH	del	*APC*
Kasumi-9	BCP-ALL	19	M	JP						*DNAH11* LOH	del	*GLI2. MECOM*, *JAK2*
Kasumi-10	BCP-ALL	6 M	F	KR-JP	*KMT2A-MLLT1*			ITD	t(11;19)(q23;p13.3)	XXY		*GATA3*, *FLT3*

^a^Populations JP: Japanese, KR: Korean, HAN: Han Chinese, TW: Taiwanese

^b^Fusion gene and mutation were not detected in this study but were reported by previous studies

### Detection of fusion genes

Fusion transcripts were detected in five cell lines (Table 3, S11). A very high level of *TCF3-PBX1* expression was observed in Kasumi-2, compared with other fusions. *NUP98-RAP1GDS1* has been identified in Kasumi-5, but it was not covered as a target in the panel. Although the panel is limited to major fusion genes, these chimeras were undetected in Kasumi-3, 6, 7 and 9, which tested negative for those fusions.

### Ethnic background

Analysis of SNP associated with population groups from which the cell lines originated revealed that six of them were classified into the Japanese population and two showed association between Korean and Japanese (Table 3). The other two were related to Han Chinese populations with Korean or Taiwanese. Although the four cell lines have mixed genetic backgrounds from different ancestral populations, pedigree information is not available for these cell lines and the generation of each individual is not clear. Our data show that all donors for the 10 cell lines belong to the east-Asian population. Sequence variations detected by the Precision ID Ancestry Panel are listed in Table S12.

### Cell surface markers

Flow cytometric histograms of 20 cell surface markers are shown in Figure S3. Positive cells were calculated from the histograms and listed as a percentage in Table S13. CD33 positive cells appeared in Kasumi-1, 3, 4 and 6, distinguishing them from the other 6 lymphoid cell lines. In contrast to the 4 myeloid cell lines, five BCP-ALL cell lines, Kasumi-2, 7, 8, 9 and 10, were positive for CD19. Although CD22 and CD38 are B-cell markers, these were detected in an AML cell line, Kasumi-3. CD13 expression was reported in the original leukemia cells of Kasumi-1 [8] but was not detected in this study. CD34 is a marker expressed in hematopoietic stem cells, which was present in Kasumi-1 and 3. Because Kasumi-4, diagnosed as chronic myelogenous leukemia blast-crisis (CML-BC), was positive for the CD13 and CD33 characteristics of AML, it could be classified with the AML cells. It is noted that a NK marker, CD56, was detected in Kasumi-3 and 4. CD3 is known as a diagnostic marker for T-cell acute lymphoblastic leukemia (T-ALL), but was absent in Kasumi-5. Kasumi-5 is unique in that it expresses a B-cell marker, CD10, which had been detected during clinical examination. This would be associated with *NUP98-RAP1GDS1* [25]. Kasumi-8, 9 and 10 showed a typical expression pattern for BCP-ALL.

Expression of HLA-DR was observed in Kasumi-1, 3 and 6 when established [9, 13, 15], but was weak or negative in our data. HLA-DR is normally expressed in BCP-ALL, and very low levels of expression in Kasumi-2 and -7 indicated the presence of positive cells in subclonal populations. These data imply that loss of HLA-DR expression would occur during cell culture.

## Discussion

A series of ten Kasumi cell lines have been evaluated by SNP microarray, each having a unique genome profile. Because gross DNA changes could affect gene expression [26], our quantitative assessment of large-scale genomic changes at chromosome level provides fundamental features for each cell line. DNA size compared to normal genomes can be determined by the differences between gains and losses, corresponding to a DNA index analyzed by a flow cytometer. Although DNA index is an efficient method to determine ploidy changes in tumor cells, chromosome rearrangements involving both gains and losses cannot be accurately shown by the balance of DNA amounts. To assess CIN, we calculated the total of the altered regions and overall size (Table 2). SGA from microarray data would reflect on the amount of chromosome rearrangements and serve as an approach for the quantitation of CIN.

Complex cytogenetic profiles are strongly associated with *TP53* mutations [27], which cause CIN [28] and lead to poor prognosis in leukemia [29]. *TP53* mutations occur during the later development of leukemic cells and promote disease progression [30]. Although *TP53* alterations can be specified by DNA sequencing, CIN is described qualitatively. Among the 10 cell lines, a high level of SGA, more than 10% of total DNA, is marked in Kasumi-1 and 6 (Fig. 1b), which were established from samples taken when the patients relapsed [8, 14]. These two cell lines exhibit *TP53* pathogenic mutations, implying that the extent of tumor progression accompanied with *TP53* deficiencies could be estimated from SGA. This will be examined in clinical cases at different phases and SGA could be applied to other cancer types.

Analysis of clinical samples by whole-genome or whole-exome sequencing reveals that AML genomes have the fewest mutations compared with other adult cancers [28]. A mutation in *CEBPA*, which is not included in the Oncomine™ Myeloid Research Assay, has been reported in Kasumi-6 [14], but it is one of the five genes often mutated in AML [28]. *GATA-2* mutation was detected in Kasumi-4, derived from childhood CML in a patient without Down syndrome. It is suggested that mutations in *GATA-2* are involved in acute myeloid transformation in CML [31], which could be applicable to Kasumi-4 characterized by CD13 and CD33 expression. Kasumi-4 could be a representative model to explore a possible role of *GATA-2* mutation and *MECOM* activation in leukemogenesis. *FLT3* mutations are often found in AML using clinical samples [32] and cell lines [33], but *FLT3-ITD* was identified in a childhood BCP-ALL cell line, Kasumi-10, which would be a distinctive feature. Because there are few cell lines accompanied with normal samples as tumor-normal pairs, this study employed normal cell lines as controls in the sequence data analysis using a custom panel. However, several variants have been detected, corresponding to differences in the hg19 reference, which cannot be clearly distinguished between SNPs and pathogenic variants. In addition, mutations which occurred during in vitro cell culture have not been identified and excluded. Our variant data can provide candidate causative genes, which would be assessed by future studies using clinical samples.

Most fusion genes in leukemia are repeatedly found [34, 35], allowing targeted sequencing to detect fusions at high rates. Alternative fusion isoforms can be resolved by targeted RNA sequencing with the expression levels [36]. Kasumi-10 has two isoforms of *KMT2A-MLLT1* with similar expression levels. Targeted sequencing could work as a first screening of major fusion genes, and minor or novel chimeras could be discovered by whole RNA sequencing. *NUP98-RAP1GDS1* is a recurrent fusion gene in T-ALL [25], which has not been identified in cell lines [20, 37], suggesting that Kasumi-5 is a unique model of T-ALL in this fusion.

Homozygous deletion of both *p16* and *p15* genes at 9p21 are frequently observed in T-ALL, but few mutations were identified in these genes [38]. Analysis of T-ALL reported that *p16* inactivation was caused not only by deletions but also by methylation [39]. SNP microarray in Kasumi cell lines revealed that deletions involving *p16* varied between samples with additional changes in other 9p regions (Figure S2). Because all four cell lines with 9p changes have *p16* alteration, instability of 9p regions would be associated with rearrangements at the *p16* locus.

Diversity is found not only in leukemia genomes but also in human populations. It is reported that sensitivity to chemotherapy is sometimes different between ethnic groups [40]. SNP analysis allows us to identify ethnic origin, which has been introduced in forensic and anthropological sciences. This has been applied to cancer cell lines, which classified them into six ancestral origins [41, 42]. Kasumi cell lines have been established from patients admitted to a hospital in Japan. Such cell lines are usually described as Japanese-derived. However, analysis of genetic ancestry may reveal different ethnic origin. Ancestry information which reflects on genetic background could be taken into account to provide precise treatment for each patient.

In contrast to solid tumors, hematopoietic samples are readily available from bone marrow or peripheral blood, allowing diagnosis to be made by direct examination of leukemic cells. Karyotyping can be achieved by a short-term cell culture of these samples, which led to the discovery of disease-specific translocations such as *BCR-ABL1* in CML [43] and *KMT2A-MLLT3* in ALL [44]. Experimental research on leukemia has been also undertaken using clinical samples obtained from patients. However, a large amount of samples from the same individual are not easily obtained for further analysis. Because Kasumi cells were derived from leukemia patients with distinct features, each Kasumi cell line has the potential to be an in vitro leukemia model.

Cancer cells frequently undergo genomic changes through proliferation, known as clonal evolution, resulting in intra-tumor heterogeneity [45]. As this is observed not only in vivo but also in vitro, tumor cell lines under the same name are not always identical because of changes during cell culture. Clonal evolution of tumor cells in vitro is different from that in in vivo in terms of limited space, and causes the replacement of cell populations during serial passage [19]. This process is specific to the in vitro procedure of subculture by dilutions of cells, which is unavoidable in their growth in culture dishes. Cell lines well-characterized by reproducible data can be used as references in comparison with other samples, leading to an accumulation of additional data and a robust experimental resource. Characterization of cell lines obtained from a public cell bank would assure high reproducibility. Our results provide fundamental genomic profiles, and can serve as reference data for the Kasumi cell lines.

Leukemia cell lines play a key role in drug development [46]; however, there has been a decrease in the establishment of new cell lines since the 1990s [47]. Differing from the LL-100 panel which provides a catalogue comprising of commonly-used cell lines [20], we present the genome profiles of 10 Kasumi cell lines, including five newly characterized ALL cell lines. Kasumi cell lines can be useful models characterized by mutations or fusion genes; *c-Kit* mutation (Kasumi-1), *MECOM* mutation/activation (Kasumi-3, 4 and 8), *FLT3-ITD* (Kasumi-6 and 10), *GATA-2* mutation (Kasumi-4), *C/EBPα* mutation (Kasumi-6), *NUP98-RAP1GDS1* fusion (Kasumi-5), and other well-known fusion genes (Kasumi-1, 2 and 10). Our panel of Kasumi cell lines promises to serve as a unique resource, which helps to develop a novel molecular target therapy.

## Electronic supplementary material

Below is the link to the electronic supplementary material.

Supplementary file1 (PDF 2225 kb)

Supplementary file2 (XLSX 230 kb)

Supplementary file3 (XLSX 1479 kb)

Supplementary file4 (XLSX 504 kb)
