# Supplementary material for: Kasumi leukemia cell lines: characterization of tumor genomes with ethnic origin and scales of genomic alterations
Source: Hum Cell. 2020 Mar 16;33(3):868–76. doi: 10.1007/s13577-020-00347-5 (PMC7324421; doi:10.1007/s13577-020-00347-5)
Supplement: Supplementary file 1 — Supplementary file1 (PDF 2225 kb) [file 13577_2020_347_MOESM1_ESM.pdf]

Figure legends

Figure S1. Whole genome profiles by SNP microarray

Copy number profiles and allele differences are shown above and bottom in each figure. An apparently normal female profile is observed in Kasumi-4 (D). UPD can be detected by allele differences, which show two allelic patterns compared to three patterns in normal regions.

Figure S2. Copy number alterations at significant loci

(A-C) CDKN2A(p16) region is highlighted in Kasumi-1, 7, 8 and 9, which show loss or deletions. (D) Chromosome 17 profiles of Kasumi-1, 5, 6 and 8 show LOH at the TP53 region. Other minor changes are detected in Kasumi-1 at 14q21.3(E), chromosome 15 (F), 16q23.1 (G), in Kasumi-7 at 9q21.13 (H), in Kasumi-3 at 3q26.2 and 7q22.1 (I) and in Kasumi-3 and 8 at ETV6 locus (J)

Figure S3. Flow cytometric histograms of cell surface markers

Percentages of positive cells are listed in Table S13.

Figure legends

Figure S1. Whole genome profiles by SNP microarray

Copy number profiles and allele differences are shown above and bottom in each figure. An apparently normal female profile is observed in Kasumi-4 (D). UPD can be detected by allele differences, which show two allelic patterns compared to three patterns in normal regions.

Figure S2. Copy number alterations at significant loci

(A-C) CDKN2A(p16) region is highlighted in Kasumi-1, 7, 8 and 9, which show loss or deletions. (D) Chromosome 17 profiles of Kasumi-1, 5, 6 and 8 show LOH at the TP53 region. Other minor changes are detected in Kasumi-1 at 14q21.3(E), chromosome 15 (F), 16q23.1 (G), in Kasumi-7 at 9q21.13 (H), in Kasumi-3 at 3q26.2 and 7q22.1 (I) and in Kasumi-3 and 8 at ETV6 locus (J)

Figure S3. Flow cytometric histograms of cell surface markers

Percentages of positive cells are listed in Table S13.

Chromosome 9 profiles

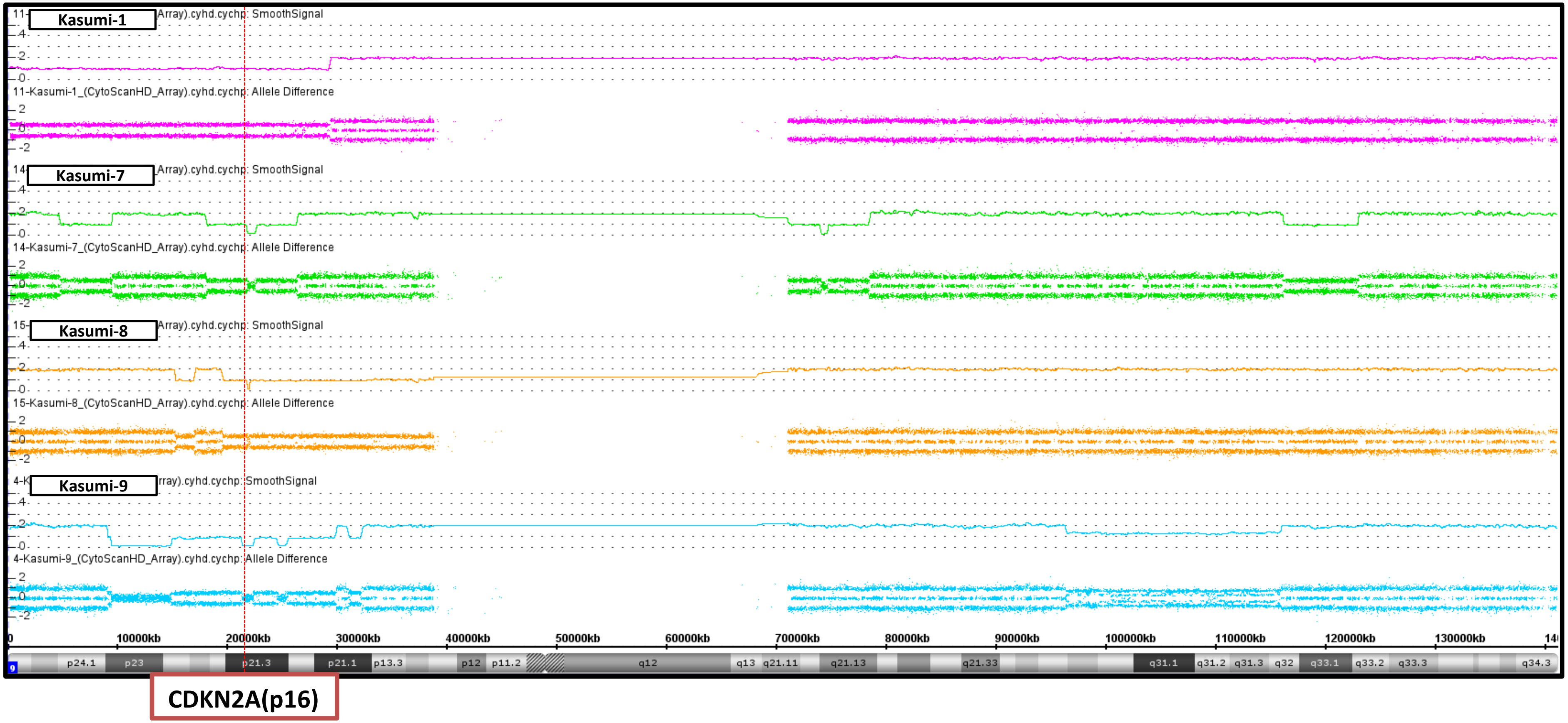

Figure S2A

9p profiles

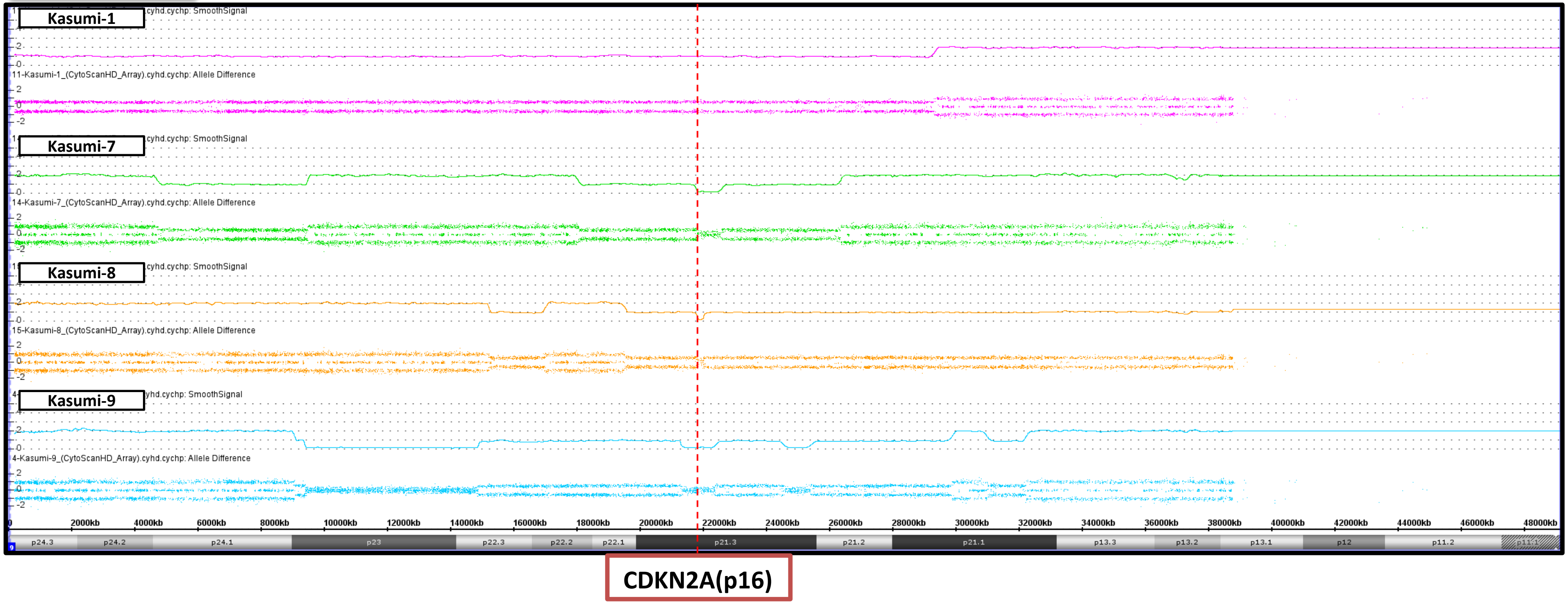

Figure S2B

9p21.3 profiles

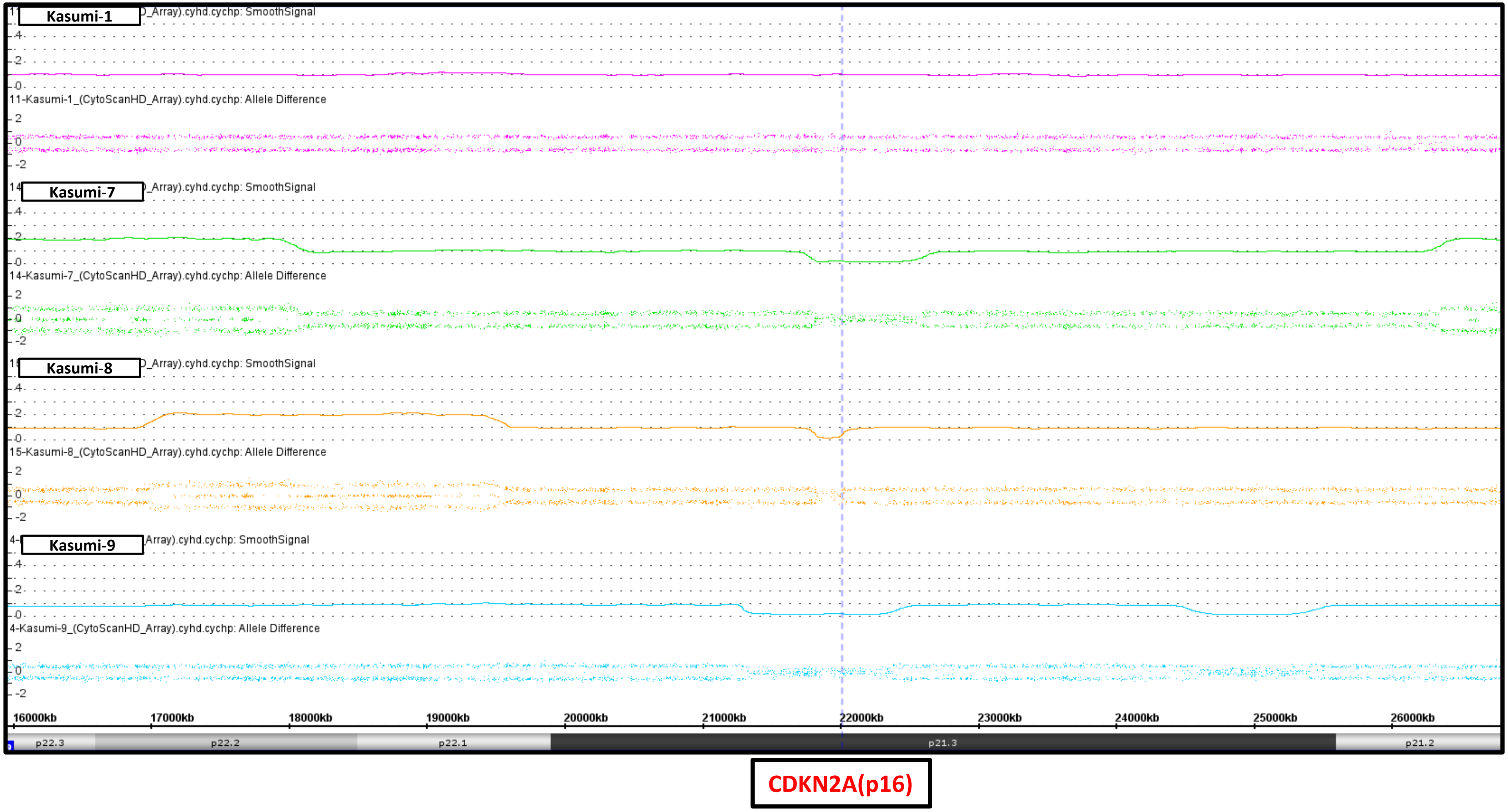

Figure S2C

Chromosome 17 profiles

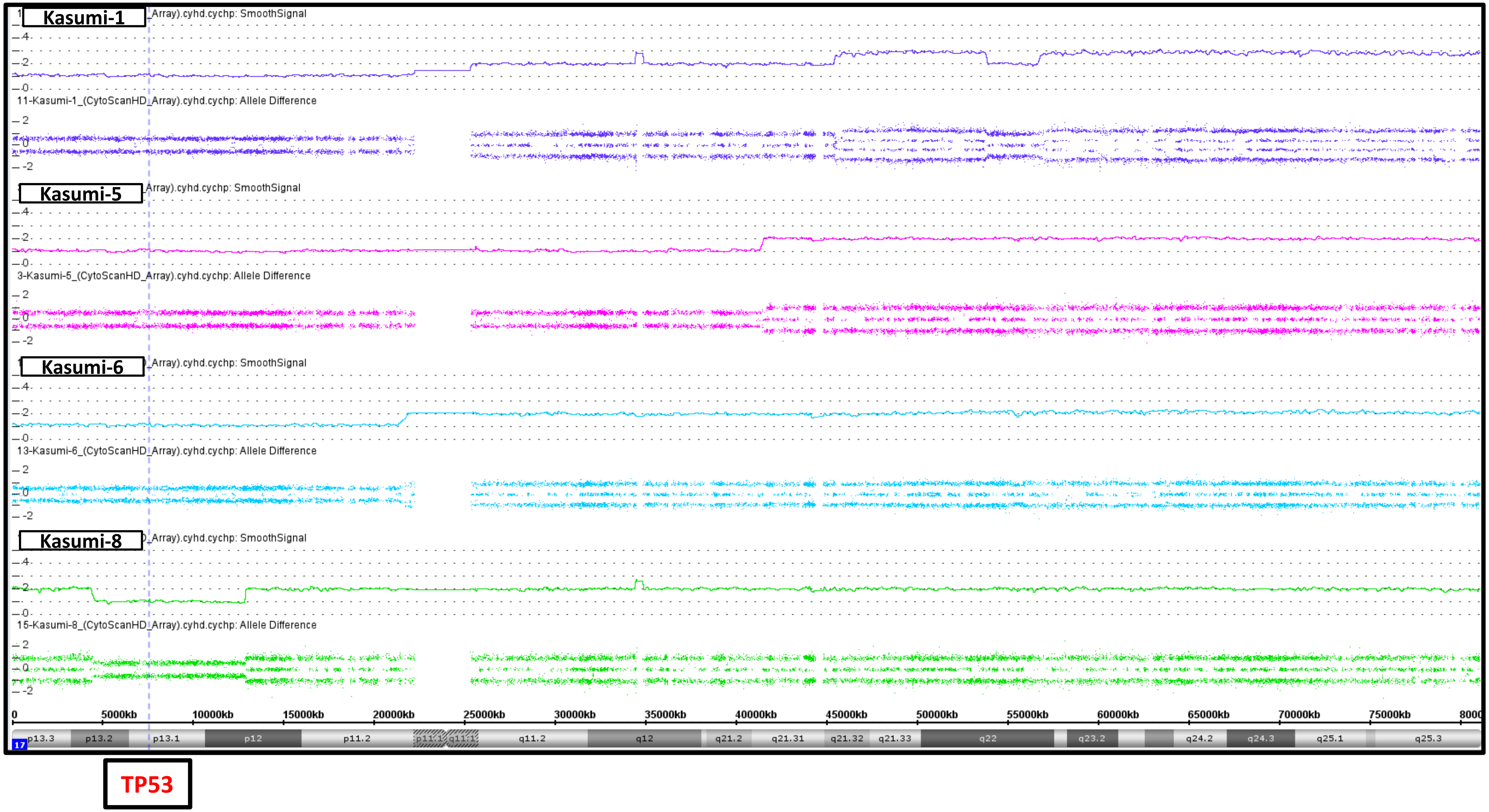

Figure S2D

Kasumi-1, 14q21.3

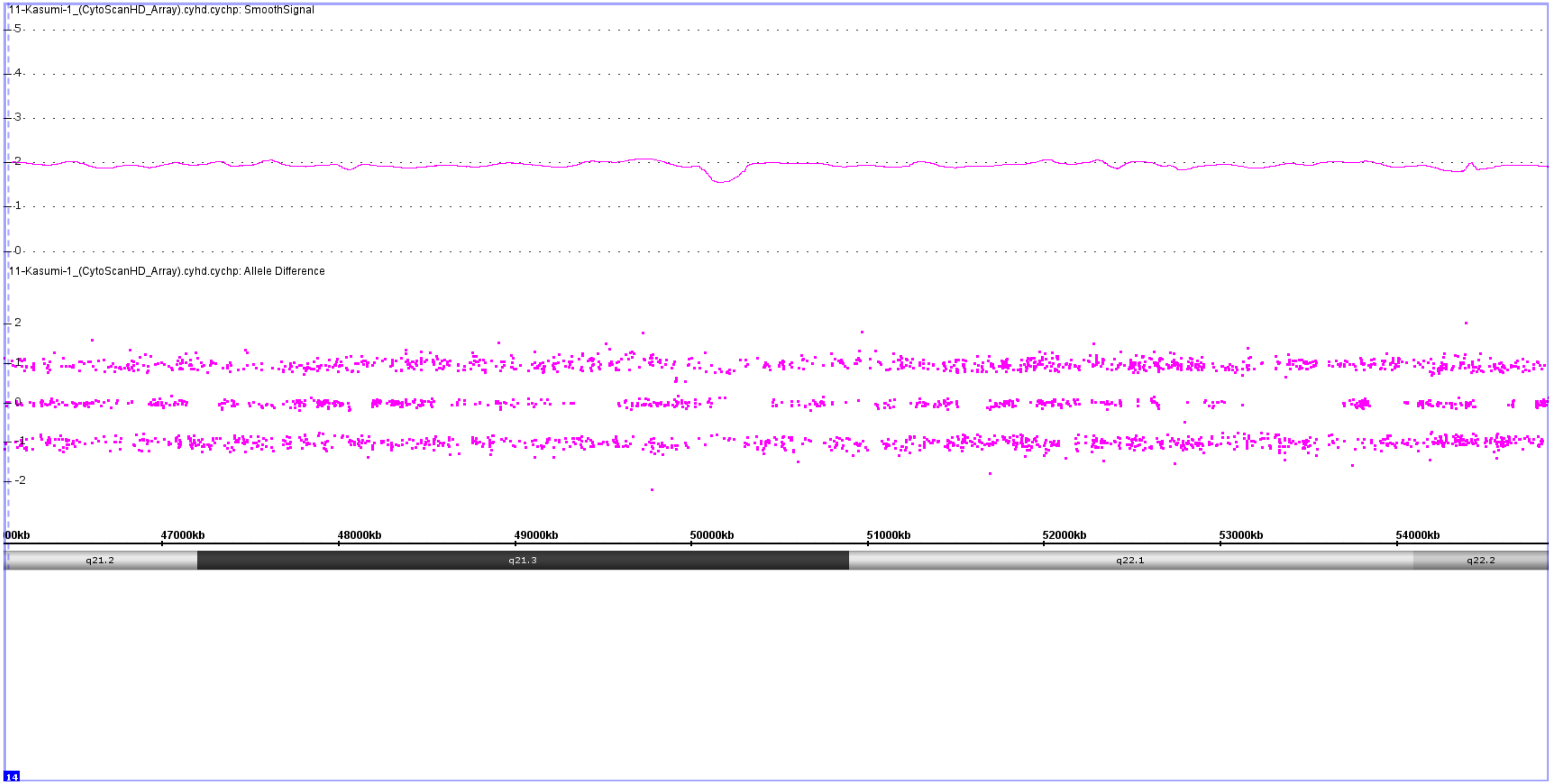

Figure S2E

Kasumi-1, chromosome 15

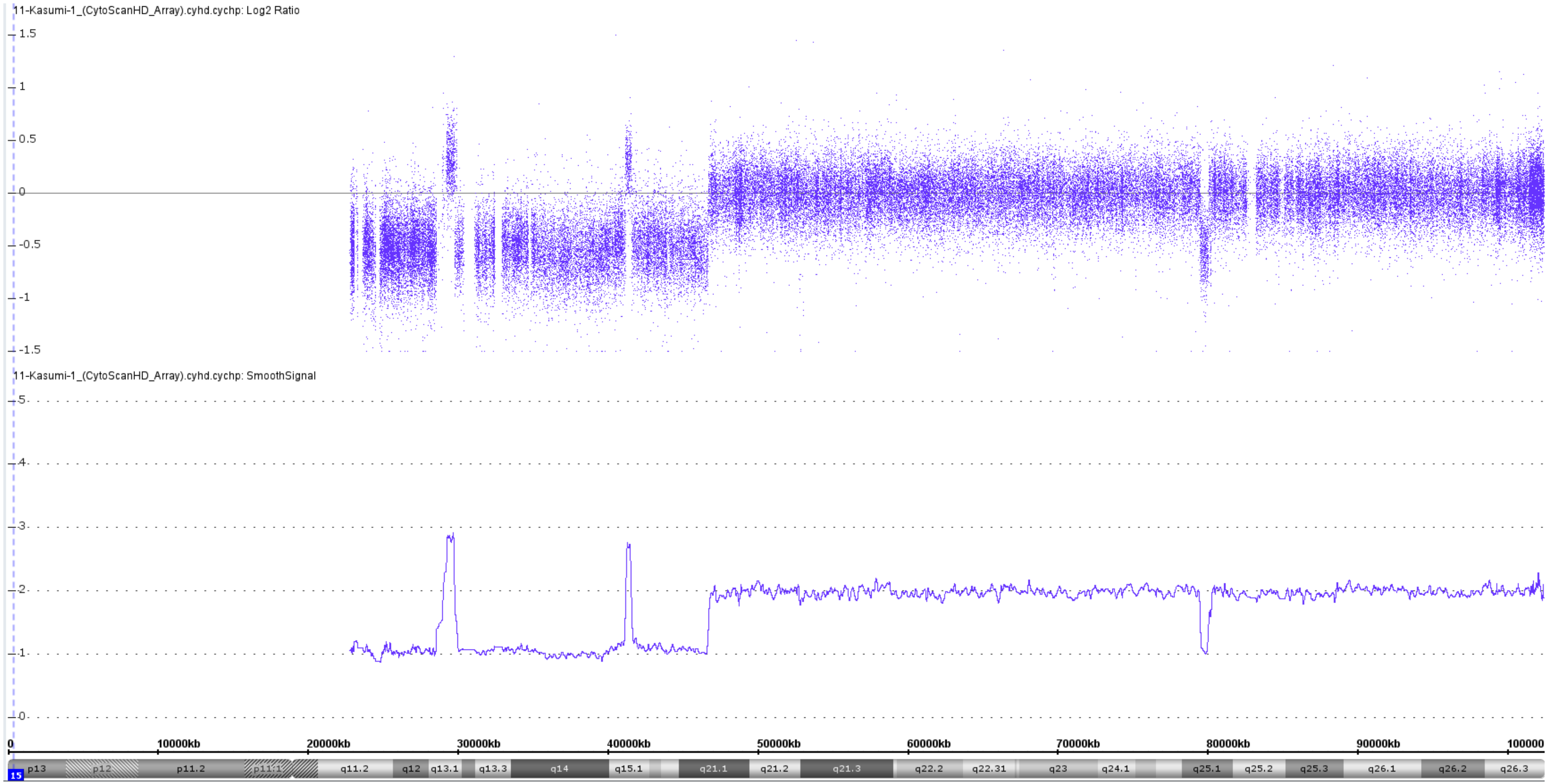

Figure S2F

Kasumi-1, 16q23.1

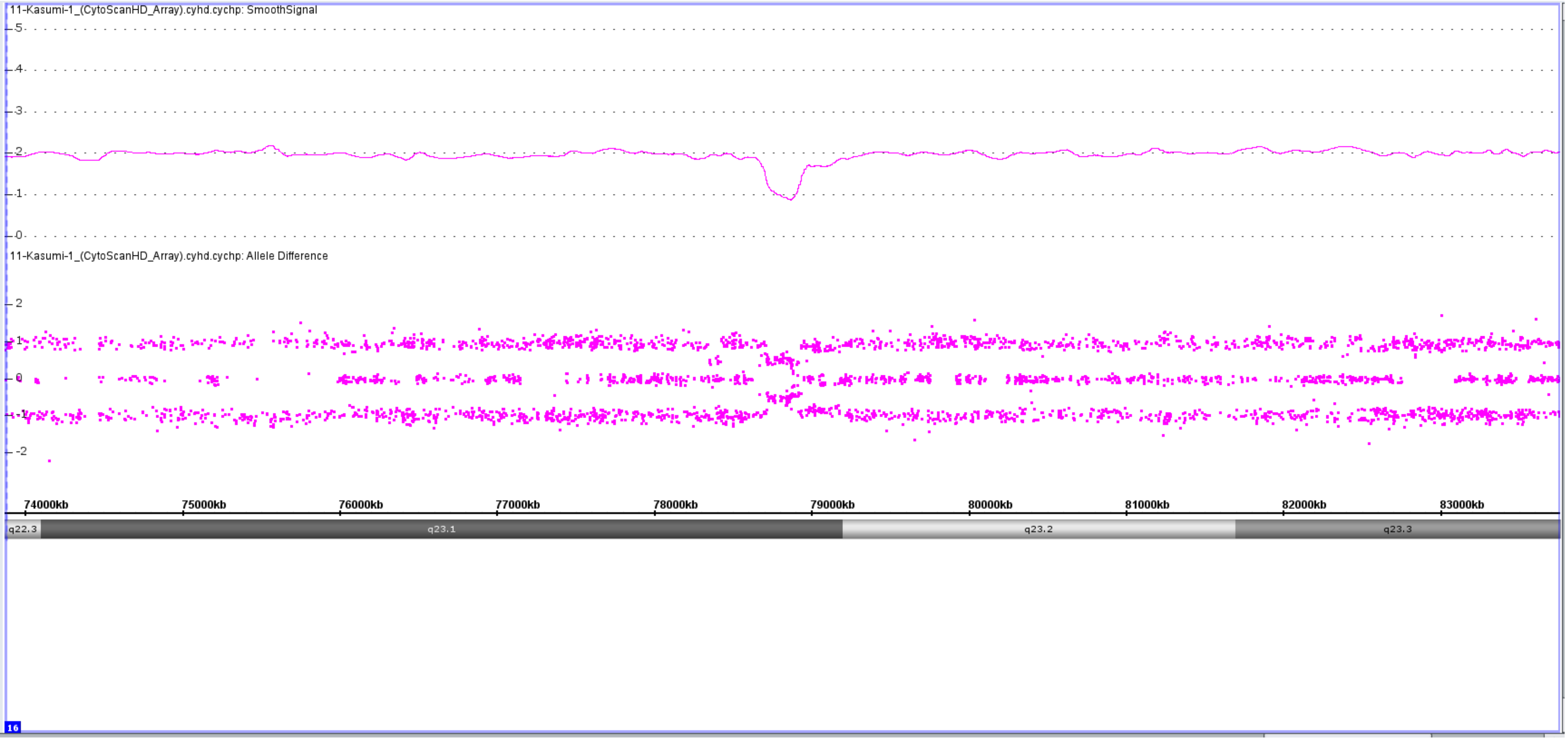

Figure S2G

Kasumi-7, 9q21.13

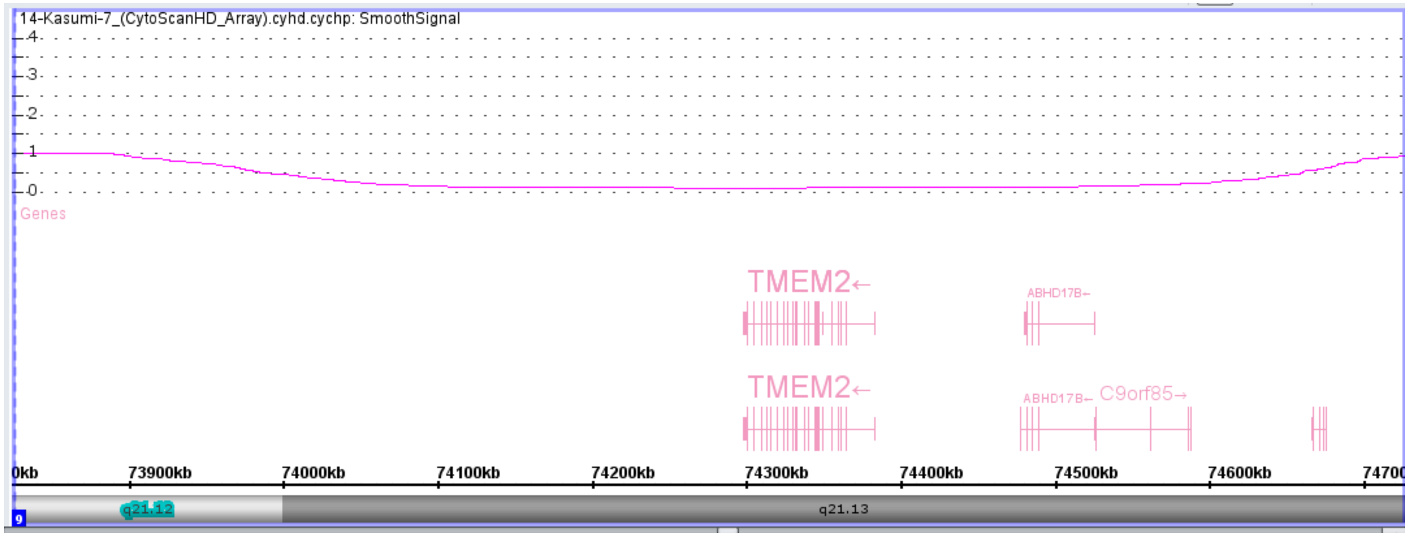

Figure S2H

Kasumi-3, 3q26.2

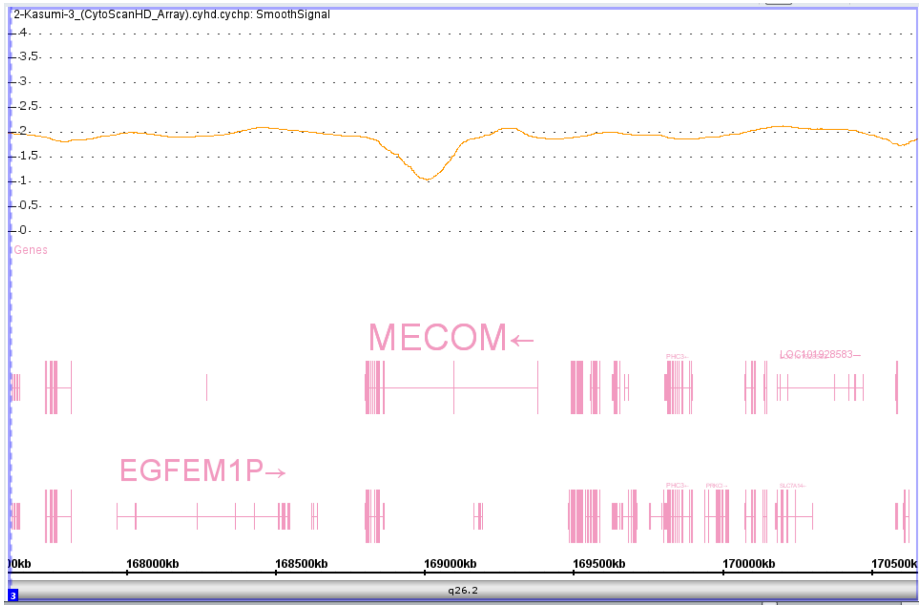

Kasumi-3, 7q22.1

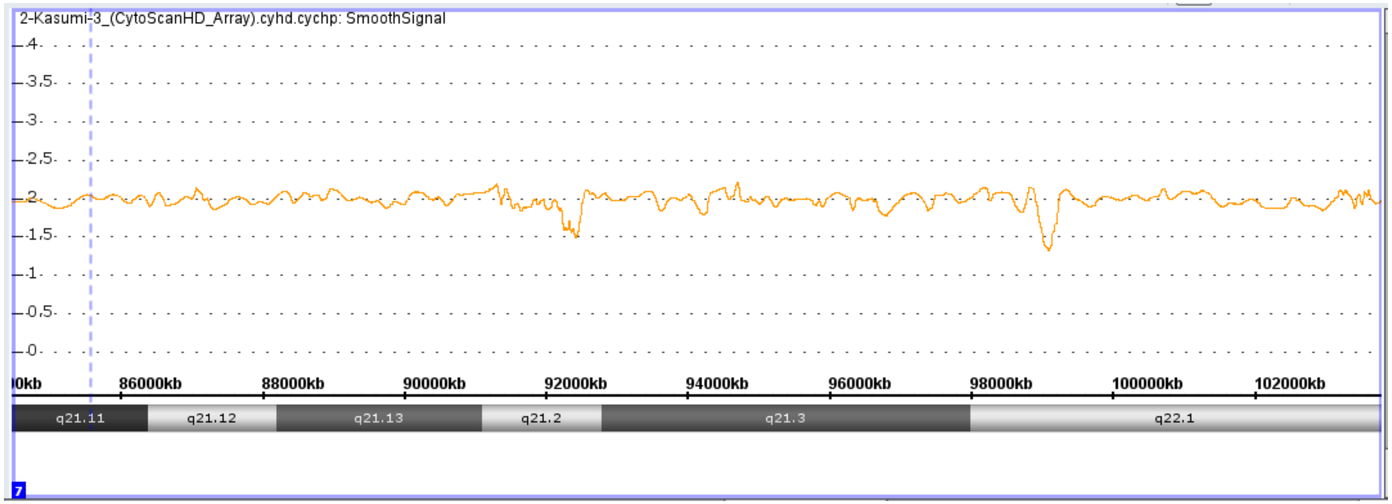

Figure S2I

12p13.2 profiles

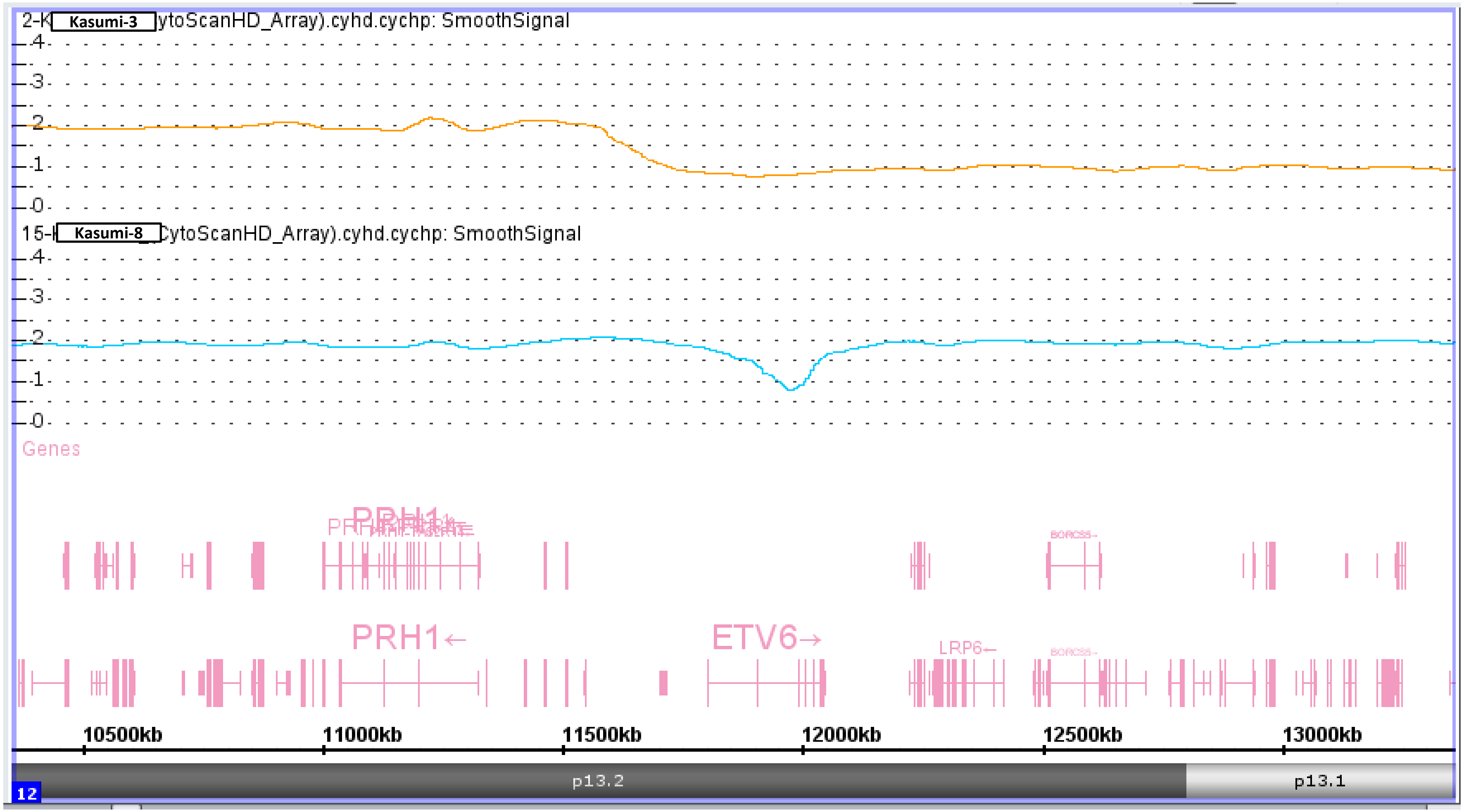

Figure S2J

Figure S3A

Kasumi-1 (JCRB1003) , P13*

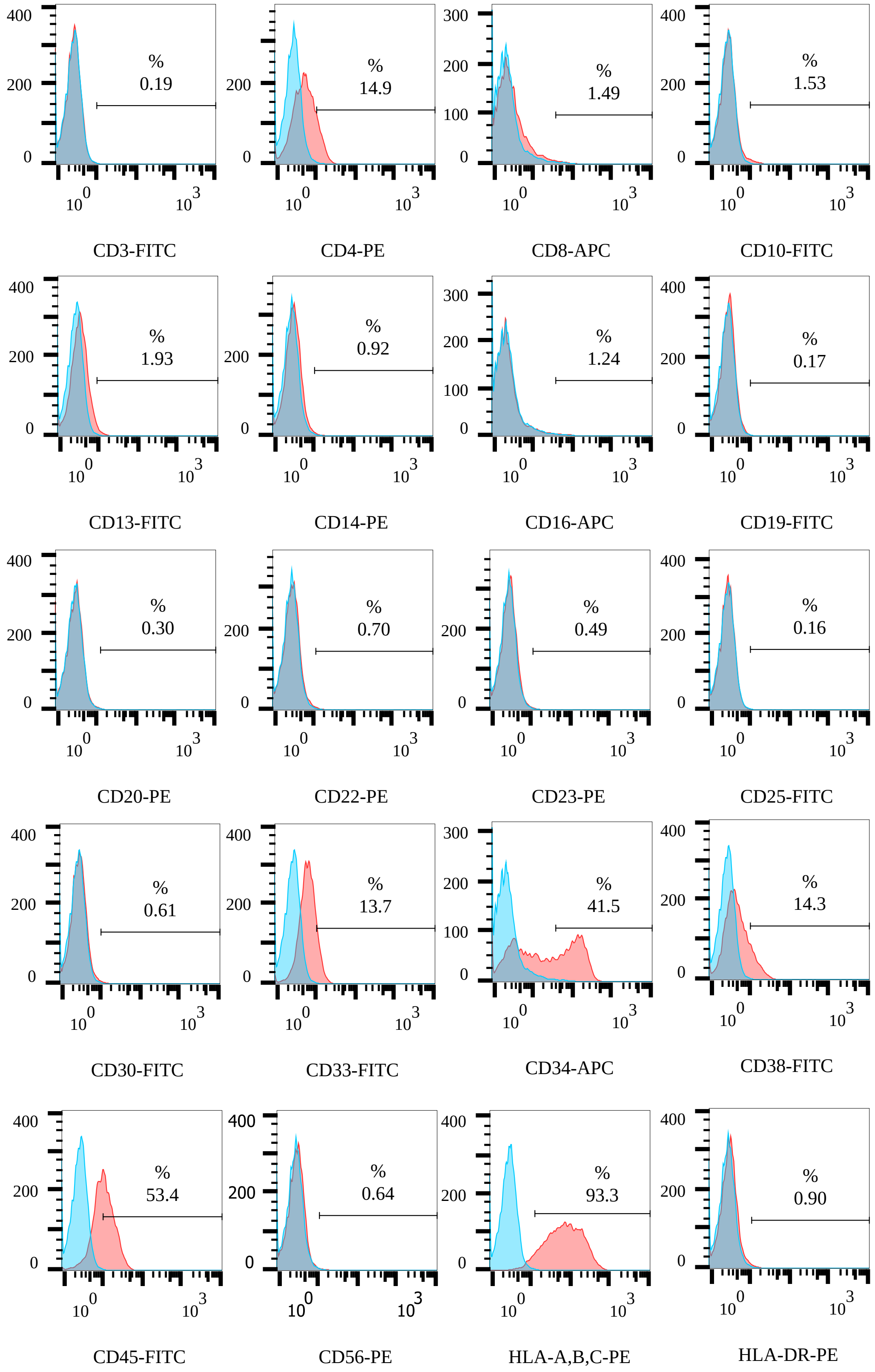

Figure S3B

Kasumi-2 (JCRB1395) , P8*

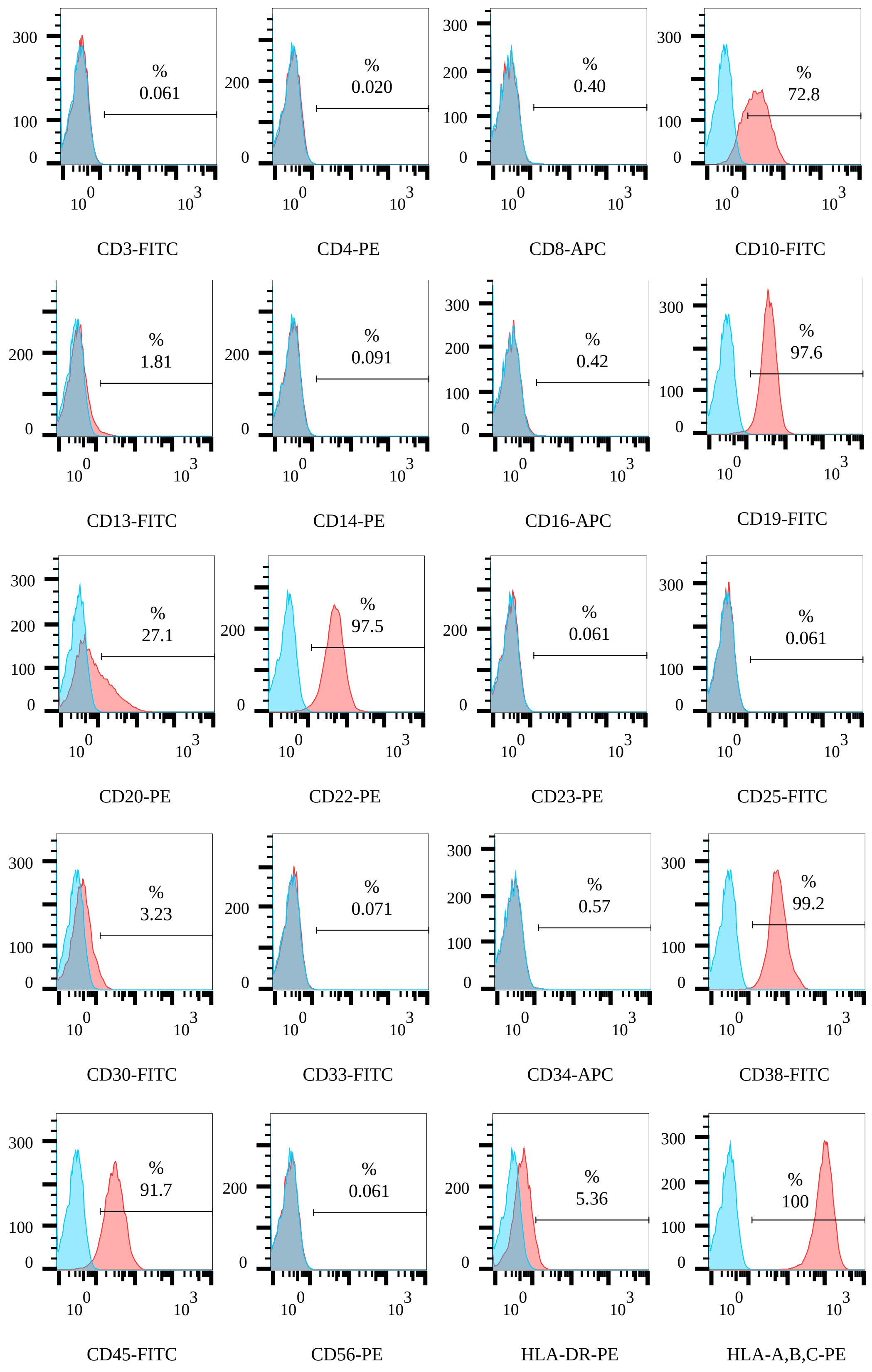

Figure S3C

Kasumi-3 (JCRB1004) , P25*

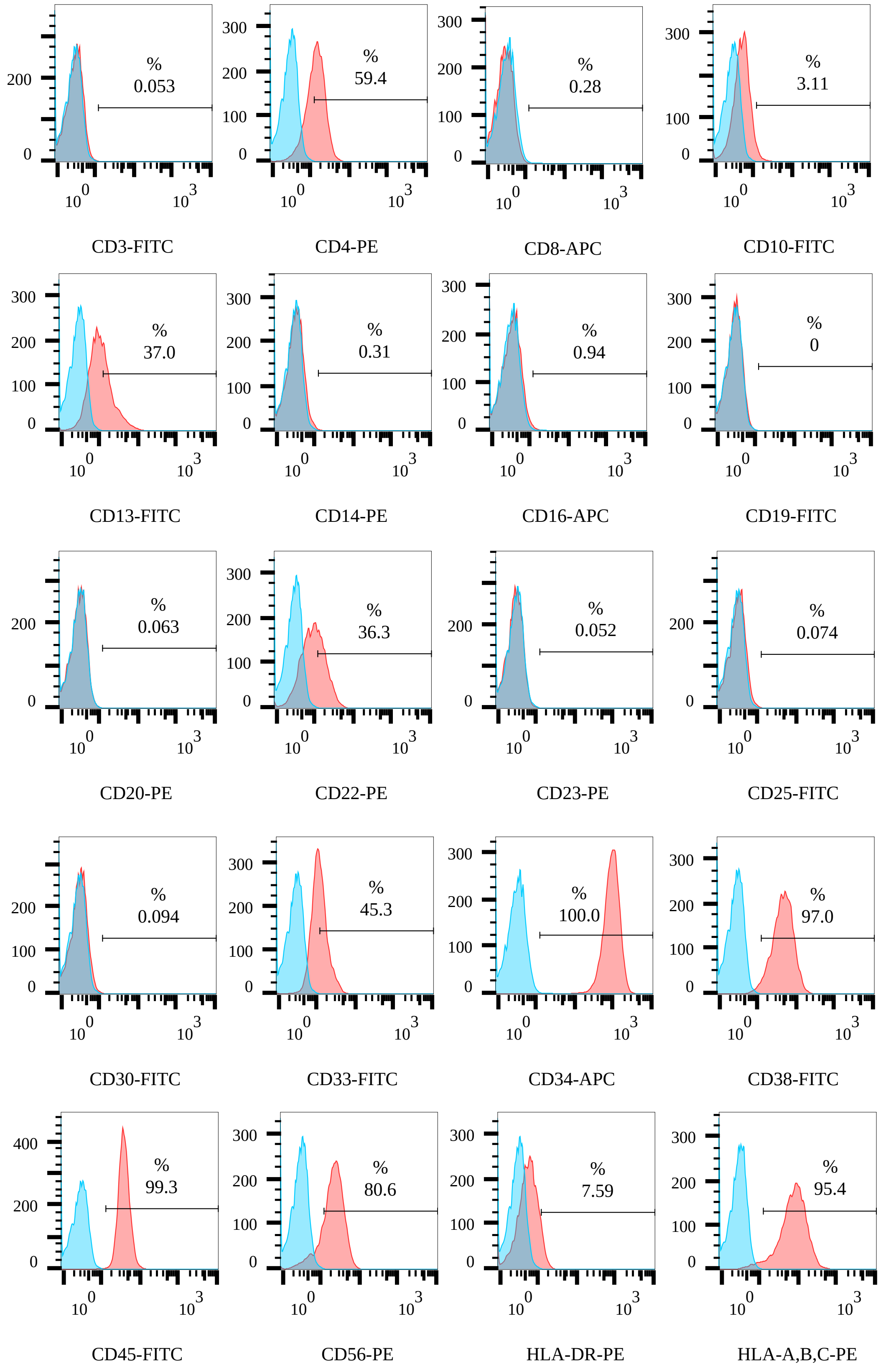

Figure S3D

Kasumi-4 (JCRB0161) , P13*

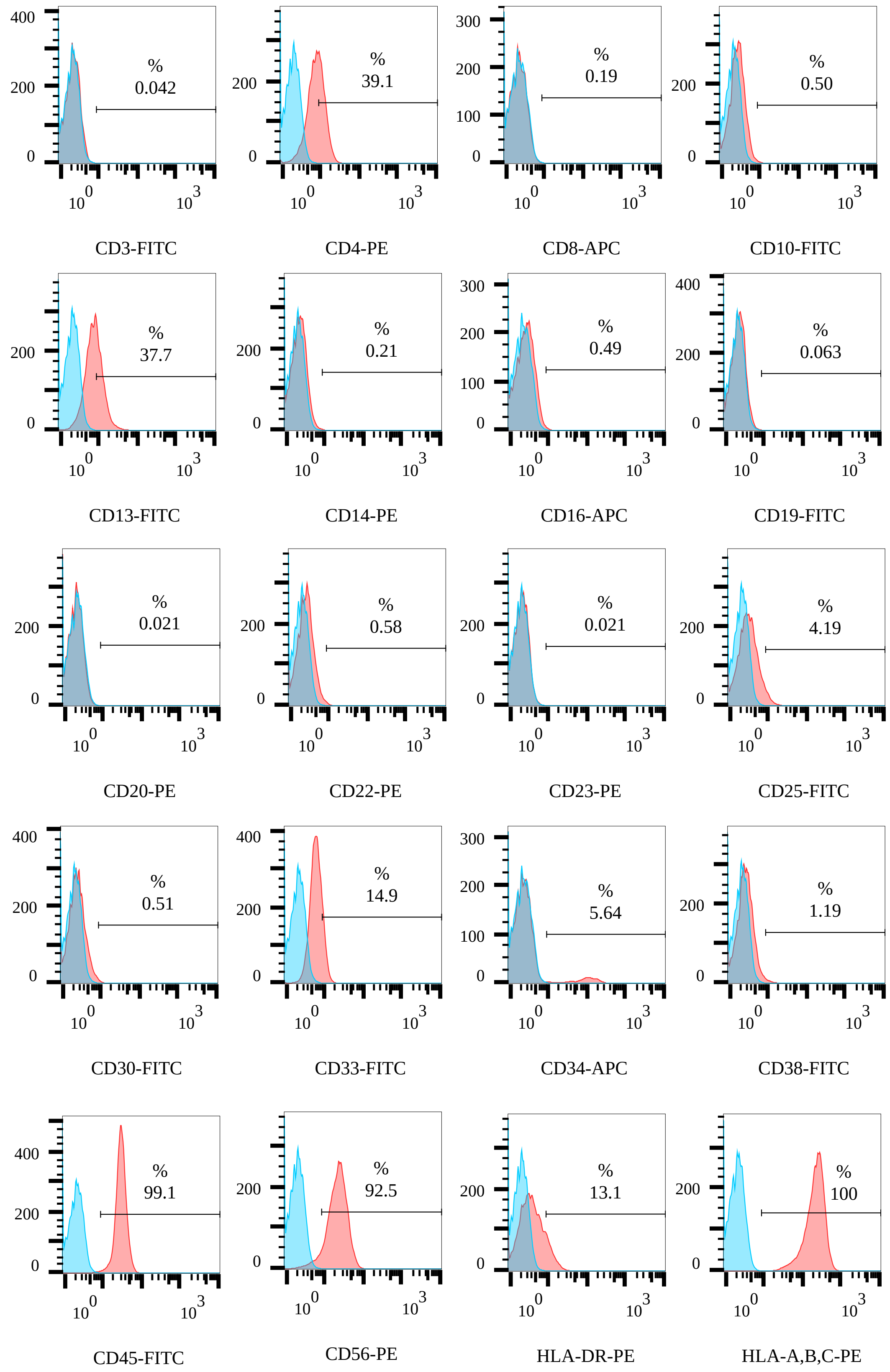

Figure S3E

Kasumi-5 (JCRB1398) , P14*

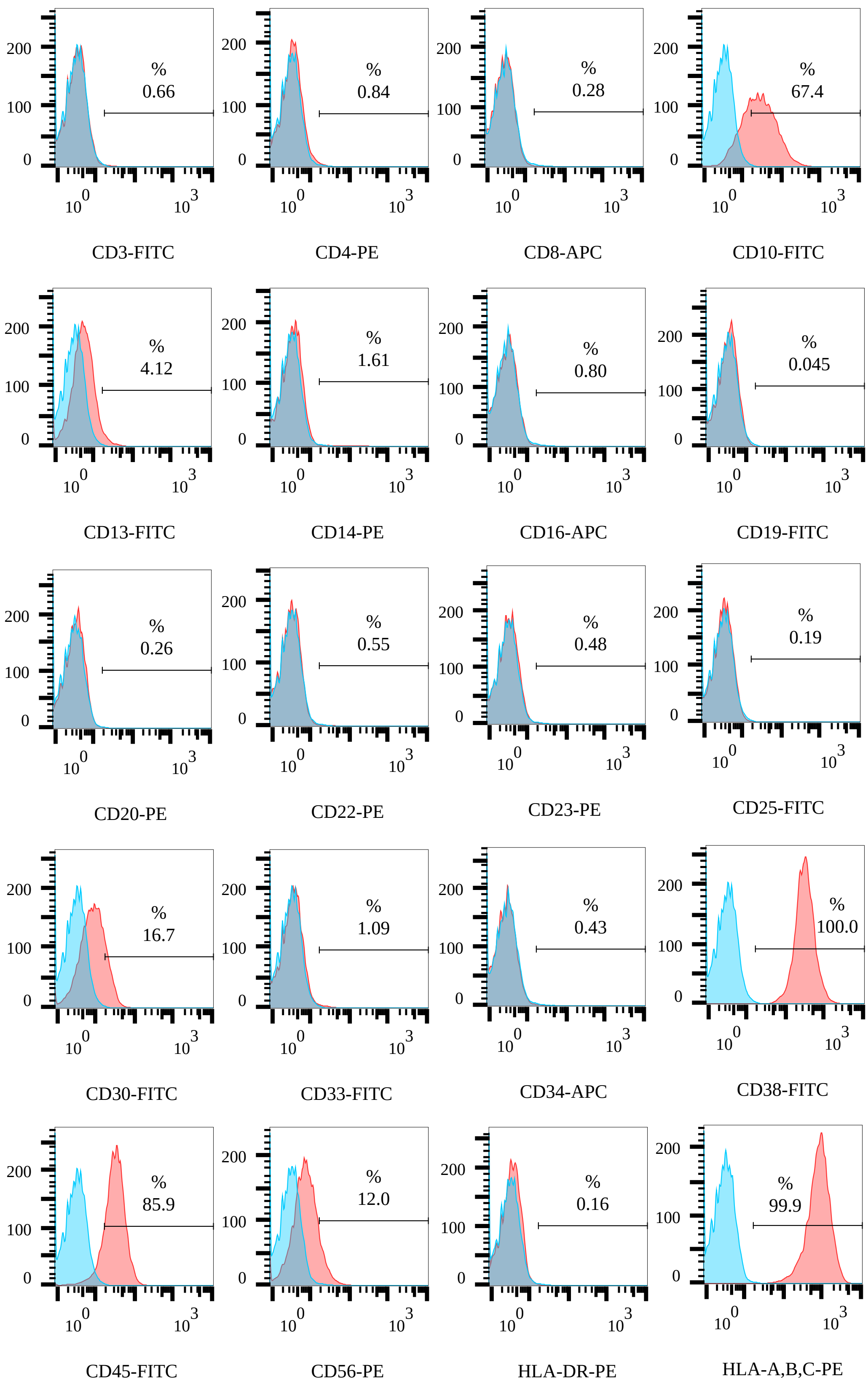

Fiure S3F

Kasumi-6 (JCRB1024) , P6*

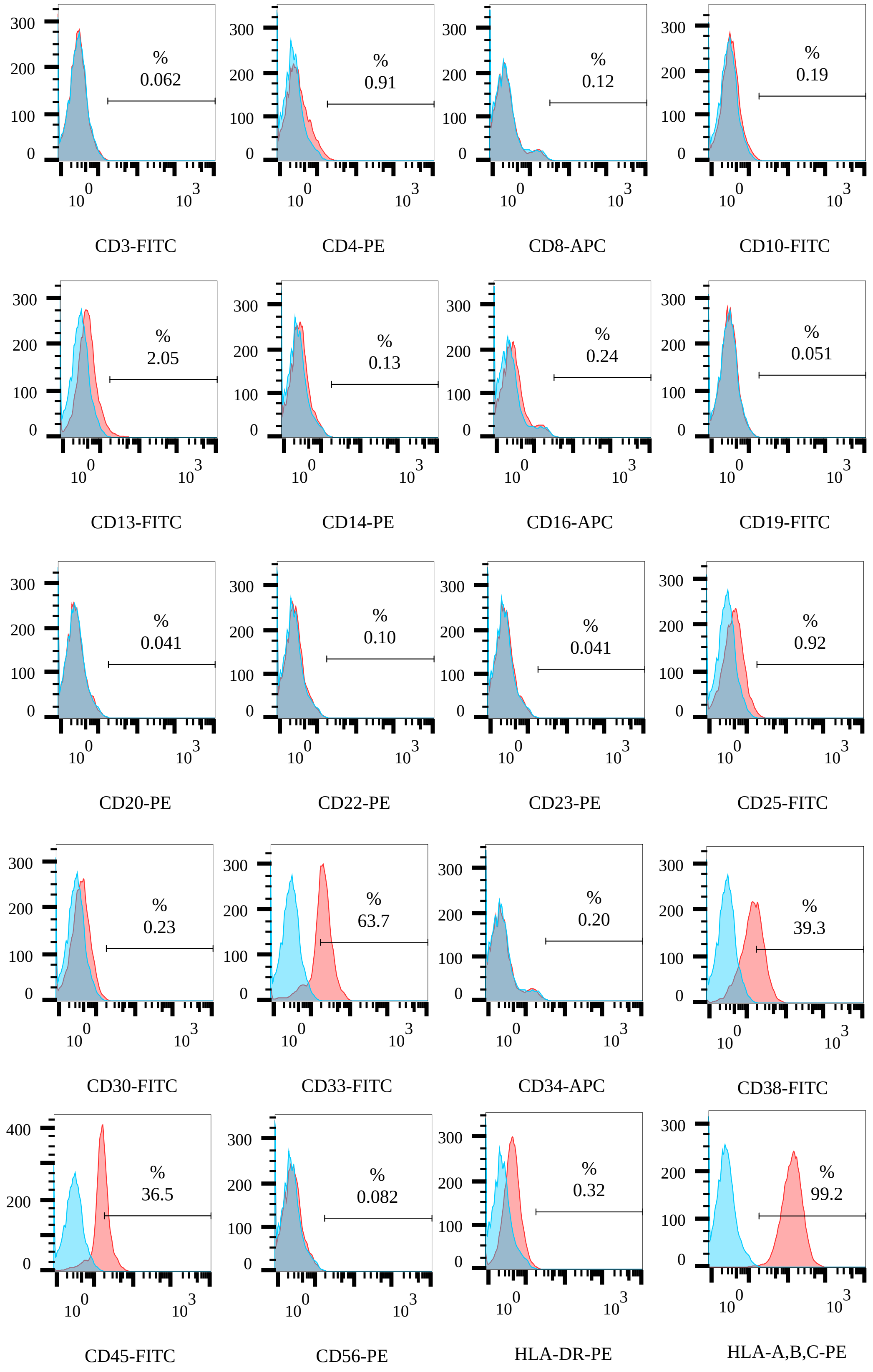

Figure S3G

Kasumi-7 (JCRB1401) , P6*

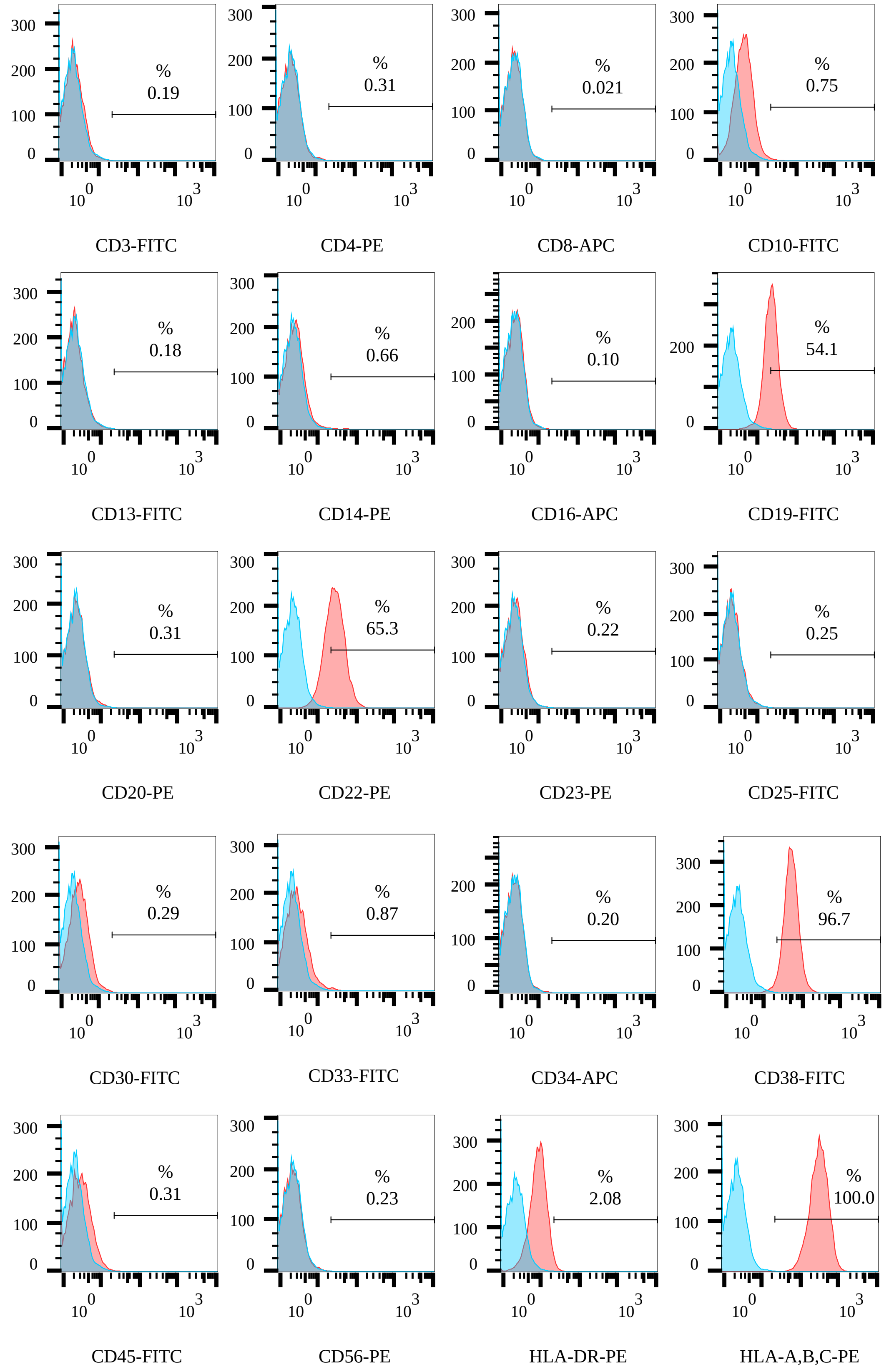

Figure S3H

Kasumi-8 (JCRB1403) P9*

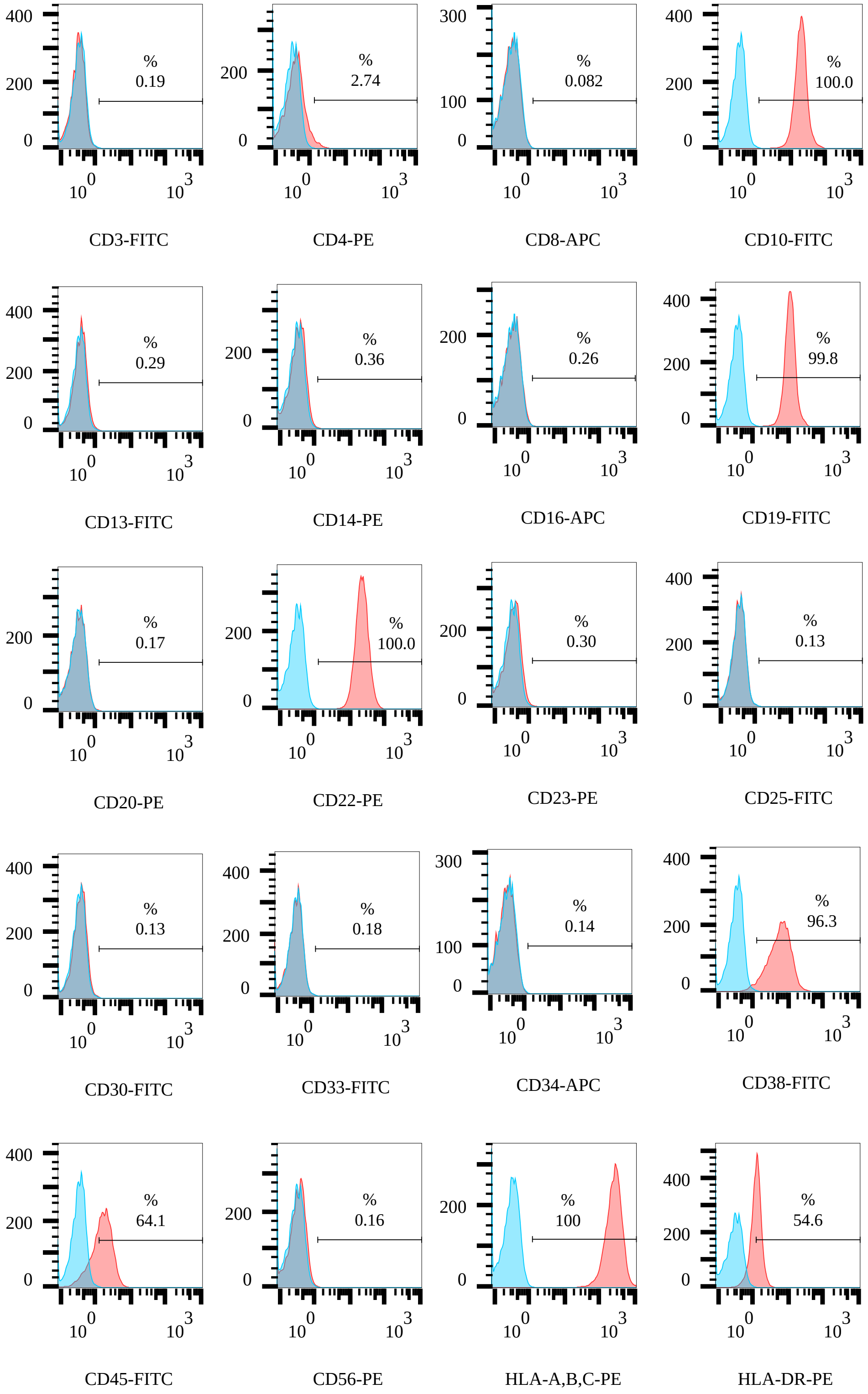

Figure S3I

Kasumi-9 (JCRB1409), P8*

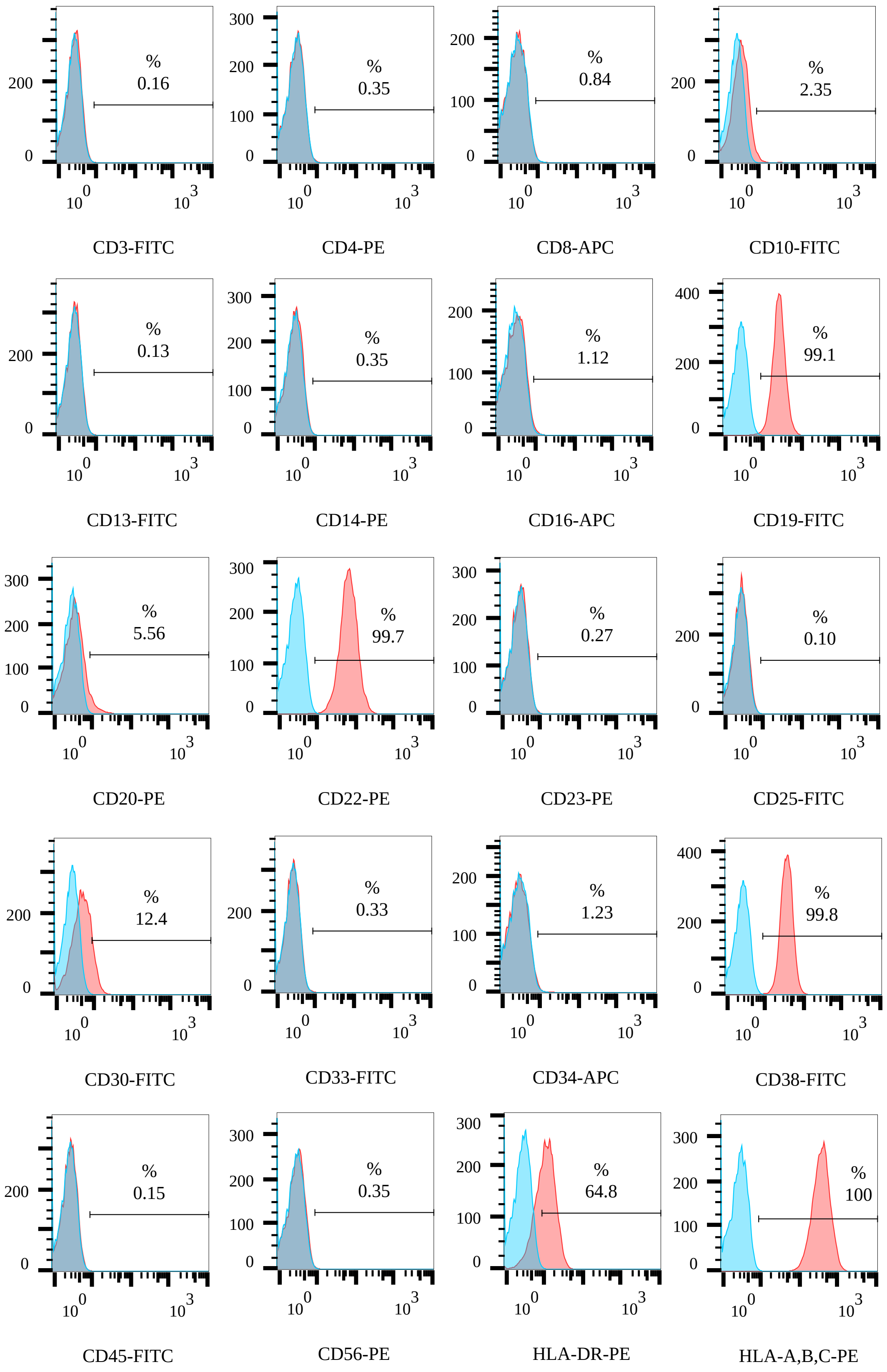

Figure S3J

Kasumi-10 (JCRB1410) , P11*

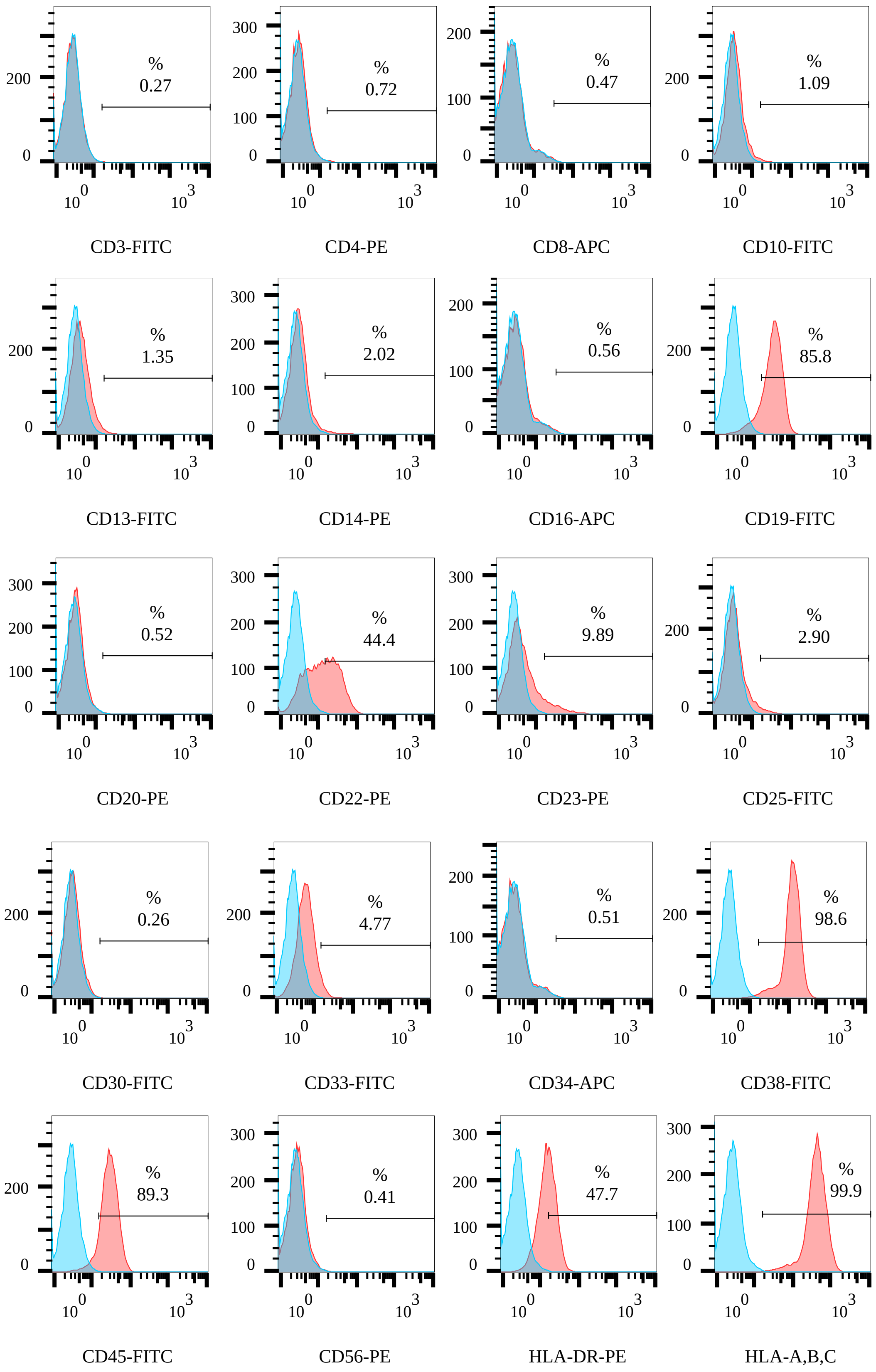

Table S1. Comparison of microarray data on Kasumi-1

Report [reference]	2q gain	5p gain	14q21.3 loss	15q25.1 loss	16q23.1 loss	Platform	Data	Source
Pedranzini et al. [15]	-	-	-	+	+	CGH Microarray, 244K	Figure 4	DSMZ
Veigaard,Kjeldsen [16]	-	-	-	-	-	Cytochip BAC array	Table 1	Not described
Peterson et al. [17]	+	+	+	-	-	Custom microarray, 180K	Data Supplement	ATCC
Present study	+	Low mosaic	Low mosaic	+	+	CytoScan HD Array, 2670K	Figure S1, S2	JCRB

Table S2. The list of FBS used for cell culture of Kasumi cell lines.

Cell Name	Cell No.	LOT No.	Passage Number*	FBS		
				Manufacturer	Catalog #	Lot #
Kasumi-1	JCRB1003	03232010	P8*	GIBCO	10099	632877
		09032015	P8*	GIBCO	10091	8156160
		02092018	P8*	Nichirei Bioscience (Sigma)	172012	12J396
		05112018	P8*	Nichirei Bioscience (Sigma)	172012	12J396
Kasumi-2	JCRB1395	02152011	P2*	Sigma	F0926	027K03911
Kasumi-3	JCRB1004	11172010	P15*	GIBCO	10099	632877
		12032010	P18*	GIBCO	10099	632877
		04272016	P19*	GIBCO	10091	8156160
Kasumi-4	JCRB0161	04282011	P8*	Sigma	F0926	027K03911
		01122018	P9*	Nichirei Bioscience (Sigma)	172012	12J396
Kasumi-5	JCRB1398	05172011	P10*	Sigma	F0926	027K03911
Kasumi-6	JCRB1024	10282013	P9*	Sigma	F0926	027K03911
		11222013	P14*	Sigma	F0926	027K03911
		04052017	P6*	Nichirei Bioscience (Sigma)	172012	12J396
		07022018	P5*	Nichirei Bioscience (Sigma)	172012	12J396
Kasumi-7	JCRB1401	04122011	P3*	Sigma	F0926	027K03911
Kasumi-8	JCRB1403	05312011	P6*	Sigma	F0926	027K03911
Kasumi-9	JCRB1409	06032011	P3*	Sigma	F0926	027K03911
		10252017	P7*	Nichirei Bioscience (Sigma)	172012	12J396
Kasumi-10	JCRB1410	06132011	P6*	Sigma	F0926	027K03911
		08272018	P10*	Nichirei Bioscience (Sigma)	172012	12J396

*Passage numbers accompanied with an asterisk indicate the numbers counted starting in the JCRB cell bank after the cells were deposited.

Table S3. List of target genes in the IAD178152 On-Demand panel							
Name	Chromosome	Num_Amplicons	Total_Bases	Covered_Bases	Missed_Bases	Overall_Coverage	Num_Exons
ABCB1	chr7	34	4113	4113	0	1	27
ABCG2	chr4	22	2118	2118	0	1	15
ABL1	chr9	27	3649	3649	0	1	12
ACOXL	chr2	18	2593	2593	0	1	17
ACTA2	chr10	8	1214	1214	0	1	8
AGO2	chr8	32	3530	3458	72	0.9796	19
AICDA	chr12	6	847	847	0	1	5
AKT1	chr14	17	1573	1573	0	1	13
ALK	chr2	40	5153	5153	0	1	29
ALOX5	chr10	25	2725	2725	0	1	14
ANXA4	chr2	12	1566	1566	0	1	12
ANXA5	chr4	13	1563	1563	0	1	12
APC	chr5	61	8857	8857	0	1	16
AQP9	chr15	10	1188	1188	0	1	6
AR	chrX	20	2873	2873	0	1	9
ARHGEF12	chr11	52	6685	6642	43	0.9936	41
ARID5B	chr10	27	4121	4121	0	1	11
ARL11	chr13	4	641	641	0	1	1
ARNTL	chr11	19	2062	2062	0	1	17
ASMTL	chrX	20	2516	2516	0	1	13
ASXL1	chr20	33	4762	4762	0	1	13
ATM	chr11	88	9791	9791	0	1	62
ATP1B1	chr1	10	1212	1212	0	1	6
ATRX	chrX	67	7829	7829	0	1	35
BAALC	chr8	4	588	570	18	0.9694	3
BACH2	chr6	18	2726	2726	0	1	4
BAX	chr19	9	1149	1149	0	1	6
BCL10	chr1	6	852	852	0	1	3
BCL2	chr18	6	853	853	0	1	2
BCOR	chrX	37	5408	5408	0	1	14
BCORL1	chrX	37	5488	5488	0	1	13
BCR	chr22	35	4966	4963	3	0.9994	23
BIRC3	chr11	18	1895	1895	0	1	8
BIRC5	chr17	7	851	750	101	0.8813	5
BMP6	chr6	10	1612	1612	0	1	7
BRAF	chr7	23	2481	2481	0	1	18
C6orf10	chr6	35	3055	3055	0	1	26
CAD	chr2	64	7118	7118	0	1	44
CALR	chr19	12	1344	1344	0	1	9
CAMK2D	chr4	27	2633	2633	0	1	21
CARD11	chr7	35	3705	3705	0	1	24
CASP7	chr10	17	1875	1875	0	1	10
CASP8	chr2	14	1813	1813	0	1	10
CBFB	chr16	6	655	655	0	1	6
CBL	chr11	23	2881	2881	0	1	16
CBLB	chr3	33	3849	3849	0	1	18

CCND1	chr11	9	938	938	0	1	5
CCR4	chr3	6	1133	1133	0	1	1
CCR7	chr17	9	1287	1287	0	1	3
CD28	chr2	6	863	863	0	1	4
CD44	chr11	24	3183	3183	0	1	19
CD5	chr11	19	1988	1988	0	1	10
CDH1	chr16	22	2809	2809	0	1	16
CDK6	chr7	10	1331	1331	0	1	7
CDKN2A	chr9	8	962	962	0	1	5
CDKN2B	chr9	4	518	518	0	1	2
CEBPE	chr14	7	946	946	0	1	2
CFLAR	chr2	18	2418	2418	0	1	12
CNOT3	chr19	25	3112	3112	0	1	17
CNR2	chr1	7	1133	1133	0	1	1
CREBBP	chr16	57	7639	7639	0	1	31
CSF1R	chr5	26	3129	3129	0	1	21
CSF2	chr5	4	635	635	0	1	4
CSF3	chr17	8	874	874	0	1	5
CSF3R	chr1	21	2857	2857	0	1	16
CTCF	chr16	17	2284	2284	0	1	10
CTLA4	chr2	6	872	872	0	1	4
CTNNB1	chr3	19	2486	2486	0	1	14
CTSZ	chr20	12	1212	1212	0	1	6
CUX1	chr7	61	7063	7054	9	0.9987	34
CYP1A2	chr15	14	1851	1851	0	1	6
CYP1B1	chr2	8	1652	1652	0	1	2
CYP2C8	chr10	16	1598	1598	0	1	10
DAPK1	chr9	40	5543	5543	0	1	25
DCAF7	chr17	10	1429	1429	0	1	8
DDC	chr7	19	2216	2216	0	1	14
DDIT4	chr10	5	799	799	0	1	2
DDX41	chr5	24	2719	2719	0	1	17
DHODH	chr16	13	1278	1278	0	1	9
DNMT3A	chr2	29	3104	3104	0	1	24
EGFR	chr7	34	4189	4189	0	1	30
ELAVL2	chr9	11	1380	1380	0	1	6
EPHB6	chr7	31	3866	3866	0	1	16
EPHX1	chr1	15	1768	1768	0	1	8
EPOR	chr19	14	1927	1927	0	1	8
ERBB2	chr17	37	4080	4080	0	1	28
ERBB4	chr2	40	4207	4207	0	1	28
ESR1	chr6	15	1874	1874	0	1	8
ETV6	chr12	12	1439	1439	0	1	8
EZH2	chr7	24	2446	2446	0	1	19
FAS	chr10	10	1098	1098	0	1	9
FBXW7	chr4	23	2692	2692	0	1	13
FGF1	chr5	5	618	618	0	1	3
FGFR1	chr8	24	2825	2825	0	1	19

FGFR2	chr10	25	2910	2910	0	1	20
FGFR3	chr4	26	2752	2752	0	1	18
FHL2	chr2	9	1090	1090	0	1	5
FIP1L1	chr4	26	2762	2720	42	0.9848	19
FLT3	chr13	28	3222	3222	0	1	24
FUS	chr16	17	1731	1731	0	1	15
FXYD6	chr11	7	588	588	0	1	6
FYN	chr6	18	2370	2370	0	1	12
GATA1	chrX	12	1292	1292	0	1	5
GATA2	chr3	13	1493	1493	0	1	5
GATA3	chr10	12	1385	1385	0	1	5
GLI1	chr12	24	3871	3871	0	1	11
GLI2	chr2	33	4891	4891	0	1	13
GNA11	chr19	12	1150	1138	12	0.9896	7
GNAQ	chr9	9	1150	1150	0	1	7
GNAS	chr20	36	4801	4801	0	1	16
GSTK1	chr7	9	1199	1199	0	1	7
GSTM1	chr1	9	1057	1057	0	1	8
GSTO1	chr10	9	1026	1026	0	1	6
GSTP1	chr11	10	983	983	0	1	7
HCK	chr20	20	2283	2283	0	1	14
HGF	chr7	24	2393	2378	15	0.9937	19
HLA-DQA1	chr6	8	968	953	15	0.9845	4
HMGA2	chr12	9	1072	1058	14	0.9869	8
HNF1A	chr12	16	2017	2017	0	1	10
HOXD4	chr2	7	868	868	0	1	2
HRAS	chr11	5	683	683	0	1	5
HSPA5	chr9	15	2365	2338	27	0.9886	8
HSPB1	chr7	7	768	768	0	1	3
ICOS	chr2	7	850	850	0	1	5
IDH1	chr2	12	1325	1325	0	1	8
IDH2	chr15	15	1469	1469	0	1	11
IFI30	chr19	10	1103	1103	0	1	7
IFNA2	chr9	3	617	617	0	1	1
IFNG	chr12	4	541	541	0	1	4
IKZF1	chr7	16	2029	2029	0	1	9
IKZF3	chr17	13	1930	1930	0	1	8
IL10	chr1	5	787	787	0	1	5
IL17A	chr6	5	618	618	0	1	3
IL19	chr1	8	948	948	0	1	6
IL2	chr4	7	662	662	0	1	4
IL4	chr5	6	662	662	0	1	4
IL6	chr7	7	839	839	0	1	5
IL7R	chr5	11	1460	1460	0	1	8
IRF4	chr6	12	1436	1436	0	1	8
IRF8	chr16	14	1681	1681	0	1	8
ITGA4	chr2	42	4581	4581	0	1	29
ITGAM	chr16	40	4962	4962	0	1	30

JAK2	chr9	31	3629	3629	0	1	23
JAK3	chr19	33	3605	3605	0	1	23
KANSL1	chr17	29	4018	4018	0	1	14
KDM6A	chrX	45	4662	4662	0	1	30
KDR	chr4	34	4371	4371	0	1	30
KEL	chr7	24	3149	3149	0	1	19
KIT	chr4	24	3141	3141	0	1	21
KMT2A	chr11	79	12279	12279	0	1	36
KMT2C	chr7	124	17686	17642	44	0.9975	59
KMT2E	chr7	49	6827	6827	0	1	25
KRAS	chr12	6	737	737	0	1	5
LMO1	chr11	8	743	743	0	1	5
LPAR1	chr9	8	1245	1245	0	1	3
LPP	chr3	16	2289	2289	0	1	9
MAP2K1	chr15	12	1292	1292	0	1	11
MECOM	chr3	33	4626	4626	0	1	18
MET	chr7	33	4427	4427	0	1	20
MGMT	chr10	6	967	967	0	1	5
MLH1	chr3	21	2461	2461	0	1	19
MLLT3	chr9	19	2310	2310	0	1	12
MPL	chr1	16	2028	2028	0	1	12
MTAP	chr9	9	1252	1252	0	1	8
MTHFR	chr1	17	2081	2081	0	1	11
MTOR	chr1	67	8220	8220	0	1	57
MTRR	chr5	18	2328	2279	49	0.979	15
MX1	chr21	22	2532	2532	0	1	13
MYB	chr6	23	3086	3086	0	1	16
MYC	chr8	9	1395	1395	0	1	3
MYD88	chr3	9	1004	1004	0	1	5
MYH11	chr16	51	6391	6391	0	1	42
NAT2	chr8	5	923	923	0	1	1
NCOA3	chr20	40	5355	5324	31	0.9942	21
NF1	chr17	74	9161	9161	0	1	58
NOTCH1	chr9	63	8008	7987	21	0.9974	34
NPM1	chr5	12	1014	1014	0	1	12
NQO1	chr16	7	1125	1125	0	1	6
NRAS	chr1	5	610	610	0	1	4
NSD1	chr5	57	8311	8311	0	1	22
NT5C2	chr10	22	2536	2536	0	1	17
NTRK3	chr15	30	3774	3774	0	1	20
OPRM1	chr6	22	2835	2835	0	1	14
P2RX7	chr12	17	2438	2438	0	1	13
PAX5	chr9	16	1707	1707	0	1	10
PDE4B	chr1	28	3577	3577	0	1	19
PDGFRA	chr4	27	3490	3490	0	1	22
PDGFRB	chr5	37	3541	3541	0	1	22
PER1	chr17	38	4973	4973	0	1	22
PER2	chr2	37	4868	4868	0	1	22

PER3	chr1	37	4683	4683	0	1	21
PHF11	chr13	13	1496	1496	0	1	10
PHF6	chrX	12	1293	1293	0	1	9
PICALM	chr11	29	3033	3033	0	1	21
PIK3CA	chr3	28	3407	3407	0	1	20
PIP4K2A	chr10	14	1721	1721	0	1	10
PLCG1	chr20	43	4196	4196	0	1	32
PMAIP1	chr18	2	265	265	0	1	2
PML	chr15	30	4208	4208	0	1	11
POLB	chr8	16	1708	1708	0	1	14
POT1	chr7	25	2655	2655	0	1	15
PRKAR1A	chr17	13	1297	1297	0	1	11
PRKCB	chr16	22	3075	3034	41	0.9867	18
PRPF8	chr17	68	9108	9108	0	1	42
PRSS1	chr7	7	794	794	0	1	5
PSIP1	chr9	23	2481	2440	41	0.9835	17
PTEN	chr10	15	1831	1831	0	1	10
PTGS2	chr1	18	1915	1915	0	1	10
PTPN11	chr12	16	1936	1936	0	1	15
PTPN2	chr18	17	2012	2012	0	1	12
PTPRJ	chr11	40	5319	5305	14	0.9974	26
PXDN	chr2	40	5590	5590	0	1	23
PYGL	chr14	25	3344	3344	0	1	20
RAC2	chr22	9	879	879	0	1	6
RAD21	chr8	19	2026	2026	0	1	13
RARA	chr17	16	2002	2002	0	1	9
RASGRP1	chr15	26	3244	3244	0	1	17
RB1	chr13	31	3057	3057	0	1	27
RBM15	chr1	18	3131	3131	0	1	3
RET	chr10	27	3577	3577	0	1	20
RGS2	chr1	6	886	886	0	1	5
RNASEH2B	chr13	14	1572	1572	0	1	12
RPL10	chrX	8	1114	1114	0	1	6
RPL5	chr1	12	1294	1294	0	1	8
RUNX1	chr21	14	1578	1578	0	1	9
RUNX3	chr1	13	1606	1606	0	1	6
SEPT9	chr17	26	3072	3072	0	1	16
SETBP1	chr18	30	5040	5040	0	1	6
SETD2	chr3	53	7905	7899	6	0.9992	21
SETDB2	chr13	23	2860	2850	10	0.9965	14
SF3B1	chr2	43	4245	4245	0	1	26
SGK1	chr6	21	2785	2785	0	1	17
SH2B3	chr12	17	1934	1934	0	1	8
SH3GL1	chr19	17	1607	1607	0	1	10
SLC19A1	chr21	18	2372	2372	0	1	7
SLC24A2	chr9	17	2486	2486	0	1	10
SMAD4	chr18	18	1769	1769	0	1	11
SMARCB1	chr22	10	1302	1302	0	1	9

SMC1A	chrX	35	4005	4005	0	1	26
SMC3	chr10	41	3944	3944	0	1	29
SMO	chr7	23	2484	2484	0	1	12
SP110	chr2	22	3220	3220	0	1	20
SPARC	chr5	11	1478	1478	0	1	9
SPI1	chr11	10	1066	1066	0	1	5
SPRY4	chr5	7	1049	1049	0	1	2
SRC	chr20	13	1721	1721	0	1	11
SRSF2	chr17	6	686	686	0	1	2
SRY	chrY	6	625	625	0	1	1
STAG2	chrX	46	4137	4117	20	0.9952	33
STAT3	chr17	25	2543	2543	0	1	23
STAT5B	chr17	20	2544	2501	43	0.9831	18
STK11	chr19	11	1392	1392	0	1	9
SVIL	chr10	55	6995	6995	0	1	35
SYNGR1	chr22	12	1197	1197	0	1	6
TAL1	chr1	8	1146	1146	0	1	3
TBL1XR1	chr3	18	2245	2245	0	1	14
TCF3	chr19	26	3142	3142	0	1	19
TERT	chr5	29	3559	3559	0	1	16
TET2	chr4	37	6188	6188	0	1	9
TFRC	chr3	26	3003	2993	10	0.9967	18
TGM6	chr20	24	2771	2771	0	1	13
THBD	chr20	10	1738	1738	0	1	1
TLX1	chr10	8	1037	1037	0	1	4
TNFRSF11A	chr18	17	2351	2351	0	1	10
TNFSF10	chr3	8	1212	1212	0	1	6
TNFSF11	chr13	10	1204	1204	0	1	5
TOP2A	chr17	50	6346	6338	8	0.9987	35
TOPBP1	chr3	51	5919	5919	0	1	27
TP53	chr17	12	1383	1383	0	1	12
TP63	chr3	22	2360	2360	0	1	16
TP73	chr1	25	2650	2650	0	1	14
TRH	chr3	6	829	829	0	1	2
TRIO	chr5	96	12144	12144	0	1	57
TRPV5	chr7	23	2940	2940	0	1	15
TSC2	chr16	45	5834	5834	0	1	41
U2AF1	chr21	10	880	880	0	1	9
UMPS	chr3	12	1503	1503	0	1	6
VDR	chr12	14	1534	1534	0	1	10
VHL	chr3	4	672	672	0	1	3
VPREB1	chr22	4	549	549	0	1	2
VSIG4	chrX	13	1736	1736	0	1	9
WHSC1	chr4	38	5270	5226	44	0.9917	22
WT1	chr11	13	1674	1674	0	1	11
XRCC1	chr19	22	2752	2752	0	1	17
ZBTB16	chr11	13	2082	2082	0	1	6
ZRSR2	chrX	17	1559	1559	0	1	11

Table S4. Filter settings for variant analysis

Allele Frequency	>= 0.1
Allele Read-Count	>= 10
Filtered Coverage	>= 20
PValue	<= 0.01
Minor Allele Frequency	<=1.0E-6
UCSC Common SNPs	Not In
Location in	exonic
Variant Effect in	unknown,<br>missense,<br>nonframeshiftInsertion,<br>nonframeshiftDeletion,<br>nonframeshiftBlockSubstitution,<br>nonsense,<br>stoploss,<br>frameshiftInsertion,<br>frameshiftDeletion,<br>frameshiftBlockSubstitution

Table S5. Cell surface markers used for flow cytometry

CD3	mIgG1	FITC	DAKO	F0818
CD4	mIgG1	RPE	Miltenyi Biotec	130-091-231
CD8	mIgG1	APC	DAKO	C7227
CD10	mIgG1	FITC	DAKO	F0826
CD13	mIgG1	FITC	Beckman coulter	IM0778U
CD14	mIgG1	RPE	Biolegend	325606
CD16	mIgG1	APC	Biolegend	302012
CD19	mIgG1	FITC	Beckman coulter	A07768
CD20	mIgG2a	RPE	Beckman coulter	IM1451
CD22	mIgG1	RPE	DAKO	R7061
CD23	mIgG1	RPE	DAKO	R7108
CD25	mIgG1	FITC	DAKO	F0801
CD30	mIgG1	FITC	DAKO	F0849
CD33	mIgG1	FITC	DAKO	F0832
CD34	mIgG1	APC	BD	555824
CD38	mIgG1	FITC	DAKO	F7101
CD45	mIgG1	FITC	DAKO	F0861
CD56	mIgG1	RPE	DAKO	R7127
HLA-ABC	mIgG2a	RPE	DAKO	R7000
HLA-DR	mIgG1	RPE	Beckman coulter	IM1639

Isotype Control

mIgG1-RPE		RPE	DAKO	X0928
mIgG1-APC		APC	DAKO	X0968
mIgG1-FITC /mIgG2a-PE		FITC/RPE	Beckman coulter	A10974

Table S6. Alterations detected by SNP microarray analysis. UPD is shown by ‘hmz’.

Cell name	Microarray Nomenclature (ISCN 2016)
Kasumi-1	arr[GRCh37] 2q21.2q33.2(134906897_204341630)x3,<br>2q33.2q37.3(204342206_242783384)x1,<br>3q13.2q13.31(112925604_114196637)x3,<br>4p12q12(46448124_54252935)x3,<br>4q12q13.1(54280345_61623168)x4,<br>7p22.3p15.3(43360_21026663)x1,<br>7p15.3p11.2(21027147_56867109)x3,<br>8q24.13q24.3(123703307_146295771)x4,<br>9p24.3p21.1(203861_29309020)x1,<br>9q21.11q34.3(71013799_141025328)x2 hmz,<br>10p15.3q26.3(142391_135427143)x3,<br>11p15.5(230615_2216183)x3,<br>12p13.33p12.3(328575_16749621)x1,<br>12p12.3(16749917_18896560)x4,<br>13q11q14.11(19436286_41586054)x1,<br>15q11.2q13.1(22770421_28958779)x1,<br>15q13.1q15.1(29904016_41159484)x1,<br>15q15.1q21.1(41585042_46668187)x1,<br>16p13.3p11.2(85880_31704526)x1,<br>17p13.3p11.1(525_22261792)x1,<br>17q21.32q22(45417166_53812743)x3,<br>17q22q25.3(56714637_81041938)x3,<br>Yq11.22q11.23(2911032_28758192)x0
Kasumi-2	arr[GRCh37] 1q23.3q44(164750227_249206020)x3,<br>2p23.1p22.3(30437776_33815335)x1,<br>3q26.33q28(179241427_191569581)x1,<br>6q13q22.32(73706990_126760994)x1,<br>7p22.3p11.2(65518_56859247)x1,<br>7q11.21q36.3(62461703_159119707)x3,<br>14q21.2q21.3(45288494_48246084)x1,<br>14q32.11q32.12(90795952_94583037)x1,<br>18q21.2(48781784_50021501)x1,<br>18q22.1q22.3(66383450_69619596)x1,<br>19p13.3(260911_1505683)x1,<br>Yq11.22q11.23(21945977_28411412)x0.3
Kasumi-3	arr[GRCh37] 1p22.1q24.2 (94506190_169477574)x2 hmz,<br>2p22.2p22.1(38537295_40337861)x1,<br>2p16.3(50526394_51929660)x1,<br>3p21.31(46968022_49081686)x1,<br>4q28.2q28.3(130325593_138049181)x2 hmz,<br>5q14.2q33.3(81970366_156449748)x1,<br>5q34q35.3(160353682_180719789)x1,<br>6p25.3p24.3(184718_8932296)x2 hmz,<br>6p21.32p21.1(32194594_40702622)x2 hmz,<br>7p22.3p15.2(2391884_26518563)x1,<br>7q34q36.1(142453878_149068732)x1,<br>9q32q33.2(115117675_123845745)x1,<br>10q21.3q26.3(66237714_135427143)x3,<br>10q25.3q26.13(114938716_125649795)x3 hmz,<br>11p15.2p15.1(13625302_21620047)x2 hmz,<br>12p13.2p11.23(11649731_27103020)x1,

	12q15q24.32(67980090_127953657)x2 hmz<br>15q11.2q14(22752398_35000987)x2 hmz,<br>16p12.3p12.1(17842757_26812386)x2 hmz,<br>16p11.2(32027964_33781862)x3,<br>16q12.1q13(49695329_57395986)x2 hmz,<br>17p13.3p13.1(2997488_9177221)x2 hmz,<br>20p12.1q12(15813474_40180864)x2 hmz,<br>20q12q13.2(40180865_50663210)x1
Kasumi-5	arr[GRCh37] 3p26.3p21.31(62613_48392800)x2 hmz,<br>6q24.2q25.3(143499742_159935292)x1,<br>12p13.33p11.23(191242_26878543)x1,<br>17p13.3q21.31 (525_41399644)x1,<br>20p13p11.21 (61568_25281959)x3,
Kasumi-6	arr[GRCh37] 1p22.1p21.2(94599283_99969159)x2 hmz,<br>2p24.2q12.1(19166927_105885194)x2 hmz,<br>2q14.3q22.1(124153065_137708701)x2 hmz<br>2q31.1(169748842_176159665)x2 hmz,<br>3p21.31p21.1(47759417_53175017)x2 hmz,<br>3q24q29(144589692_197851986)x3,<br>10p15.3 q26.3 (100026_135427143)x3,<br>11p13q12.1(33966694_58598012)x2 hmz,<br>12p13.33p11.1(173786_34835837)x1,<br>13q11q22.1(19436286_74500228)x3,<br>13q22.1q34(74910513_115107733)x4<br>17p13.3p11.2(525_21555368)x1,<br>19p13.3p11(260911_24505637)x2 hmz,<br>21q11.2q22.3(15006457_48097372)x3,<br>22q11.1q13.33(16888899_51197838)x3
Kasumi-7	arr[GRCh37] 1q32.1q41(205609009_215244510)x2 hmz,<br>4q12q13.1(59030168_66080253)x1,<br>4q13.2(67652806_69051094)x1,<br>4q13.2q13.3(70465736_72651433)x1,<br>4q13.3(73090506_75752089)x1,<br>4q22.2q22.3(94954025_98481870)x1,<br>8q11.1q11.21(47189058_48400130)x3,<br>9p22.2p21.2(18061139_26349225)x1,<br>9p24.1p23(4758060_9476653)x1,<br>9q21.11q21.13(70989957_78456306)x1,<br>9q32q33.2(116141808_122970210)x1
Kasumi-8	arr[GRCh37] 9p22.1q13(19516862_67983174)x1,<br>9p22.3p22.2(15227687_16986789)x1,<br>17p13.2p12(4438906_12861563)x1,<br>19p13.3(1902841_4014482)x1,<br>20q12q13.2(38759789_51606561)x1
Kasumi-9	arr[GRCh37] 5q11.1q11.2(49843246_51972210)x1,<br>5q31.3q33.2(141034183_155590271)x1,<br>9p21.1(30992687_32211763)x1,<br>9p23p21.1(9074934_29871355)x1,<br>9q22.31q32(96353484_115939209)x1.3,<br>18q21.31q21.33(55881149_60067241)x1.3,<br>19p13.11q12(16958954_29599139)x2 hmz
Kasumi-10	arr[GRCh37] 16p12.1q12.1(25490344_51698586)x2 hmz,

Table S7-1. Coverage data from DNA analysis of the Ampliseq Oncomine Myeloid panel

Sample	Mapped reads	On target	Mean depth	Uniformity	Amplicons with at least 20 reads
Kasumi-1	241,915	98.25%	462.3	86.81%	98.29%
Kasumi-2	478,591	98.45%	915.4	87.36%	98.86%
Kasumi-3	872,175	95.72%	1521.0	73.90%	95.82%
Kasumi-4	318,999	98.12%	605.8	90.33%	99.05%
Kasumi-5	419,097	98.23%	801.5	91.70%	98.86%
Kasumi-6	836,185	94.44%	1430.0	76.07%	97.15%
Kasumi-7	458,874	98.48%	877.6	91.51%	99.24%
Kasumi-8	275,409	98.58%	531.0	87.77%	98.67%
Kasumi-9	1,023,198	97.93%	1918.0	59.96%	99.05%
Kasumi-10	257,666	98.61%	495.9	90.26%	98.86%

Table S7-2. Coverage data from RNA analysis of the Ampliseq Oncomine Myeloid panel

Sample	Unaligned Reads	Mapped Fusion Panel Reads	Expression Controls Total Reads
Kasumi-1	71,190	48931	26796
Kasumi-2	18,086	12840	5997
Kasumi-3	18,362	12020	7294
Kasumi-4	40,699	30940	16367
Kasumi-5	16,237	13807	10751
Kasumi-6	106,308	53573	34084
Kasumi-7	20,414	14156	8946
Kasumi-8	36,005	22902	15106
Kasumi-9	61,322	35559	22133
Kasumi-10	29,917	20921	12979

Table S7-3. Coverage data from the Ampliseq On-Demand panel, IAD178152

On-Demand	Mapped reads	On target	Mean depth	Uniformity	Amplicons with at least 20 reads
Kasumi-2	1,143,785	94.75%	180.2	95.82%	97.42%
Kasumi-5	903,201	95.12%	142.2	95.66%	96.74%
Kasumi-7	1,031,913	95.00%	163.3	95.56%	97.00%
Kasumi-8	1,845,333	94.37%	291.0	95.38%	97.58%
Kasumi-9	3,462,158	94.33%	536.4	89.28%	97.95%
Kasumi-10	878,401	95.80%	139.4	96.03%	97.02%
SF-TY	743,458	94.18%	115.7	96.97%	97.40%
TIG-7-20	985,071	94.76%	154.0	96.73%	98.01%

Table S7-4. Coverage data from the Precision ID Ancestry Panel

HID	Mapped reads	On target	Mean depth	Uniformity	Amplicons with at least 20 reads
Kasumi-1	158,769	91.21%	904.0	59.94%	85.45%
Kasumi-2	66,572	93.96%	367.7	54.55%	73.33%
Kasumi-3	75,675	94.19%	429.9	53.17%	75.76%
Kasumi-4	67,830	94.03%	381.1	61.44%	79.39%
Kasumi-5	71,842	94.09%	405.6	57.28%	76.38%
Kasumi-6	50,715	92.27%	296.8	56.33%	67.88%
Kasumi-7	53,975	92.87%	317.2	58.19%	70.91%
Kasumi-8	118,925	91.66%	680.8	59.32%	84.85%
Kasumi-9	298,708	92.54%	1606.0	58.07%	90.30%
Kasumi-10	99,852	91.45%	578.8	58.12%	81.21%
SF-TY	89,490	94.20%	520.6	57.91%	80.61%
TIG-7-20	120,996	91.54%	673.1	58.92%	81.82%

Table S9. Sequence variants identified by the Oncomine Myeloid panel

	Genes	Locus	Genotype	Ref	Observed Allele	Type	Length	Gene Class	% Frequency	Array CN	Amino Acid Change	Coverage	Exon	Coding	Variant Effect
Kasumi-1	KIT	chr4:55599340	T/A	T	A	SNV	1	Gain-of-function	79.3	4	p.Asn822Lys	353	17	c.2466T>A	missense
	TP53	chr17:7577538	T/T	C	T	SNV	1	Loss-of-function	99.9	1	p.Arg248Gln	653	7	c.743G>A	missense
Kasumi-2	HRAS	chr11:534286	C/T	C	T	SNV	1	Gain-of-function	37.3	2	p.Gly13Ser	1609	2	c.37G>A	missense
Kasumi-3	ETV6	chr12:12022582	CAG/C	CAG	C	INDEL	2	Loss-of-function	21.4	1	p.Gln230fs	1990	5	c.689_690delAG	frameshiftDeletion
Kasumi-4	GATA2	chr3:128200730	A/C	A	C	SNV	1	Gain-of-function	46.2	2	p.Leu359Val	403	5	c.1075T>G	missense
Kasumi-6	WT1	chr11:32417851	TCT/T	TCT	T	INDEL	2	Loss-of-function	49.2	2	p.Arg401fs	1975	7	c.1199_1200delAG	frameshiftDeletion
	FLT3	chr13:28608257	T/TCATATTCATATTCTCTGAAATCAACGTAGAAGTACTCATTATCTGAGGAGC	T	TCATATTCATATTCTCTGAAATCAACGTAGAAGTACTCATTATCTGAGGAGC	INDEL	51	Gain-of-function	16.6	3	p.Gly583_Tyr599dup	3211	14	c.1798_1799insGCTCCTCAGATAATGAGTACTTCTACGTTGATTTCAGAGAATATGAATATG	nonframeshiftInsertion
	FLT3	chr13:28608309	C/CATATTCATATTCTCTGAAATCAACGTAGAAGTACTCATTATCTGAGGAGCC	C	CATATTCATATTCTCTGAAATCAACGTAGAAGTACTCATTATCTGAGGAGCC	FLT3ITD	51	Gain-of-function	41.6	3	p.Thr582_Gly583insGlySerSerAspAsnGluTyrPheTyrValAspPheArgGluTyrGluTyr	1480	14	c.1746_1747insGGCTCCTCAGATAATGAGTACTTCTACGTTGATTTCAGAGAATATGAATAT	nonframeshiftInsertion
	TP53	chr17:7577581	C/C	A	C,G,T	SNV	1	Loss-of-function	100.0	1	p.Tyr234Asp	1999	7	c.700T>G	missense
	STAG2	chrX:123224455	A/A	G	A	SNV	1	Loss-of-function	99.9	1	p.Trp1103Ter	697	31	c.3308G>A	nonsense
Kasumi-8	ETV6	chr12:12006443	A/AAG	A	AAG	INDEL	2	Loss-of-function	97.0	1	p.Asn138fs	430	4	c.412_413insGA	frameshiftInsertion
Kasumi-10	FLT3	chr13:28608271	T/AGGGAAATCTTCACTGAAATCAACGTAGAAGTACTCATTAT	T	AGGGAAATCTTCACTGAAATCAACGTAGAAGTACTCATTAT	FLT3ITD	41	Gain-of-function	27.3	2	p.Glu596Ter	491	14	c.1785_1785delAinsATAATGAGTACTTCTACGTTGATTTCAGTGAAGATTTCCCT	nonsense
	FLT3	chr13:28608271	T/TCTGAAATCAACGTAGAAGTACTCATTATCTGAGGAGCCGGTCAAATATTCA	T	TCTGAAATCAACGTAGAAGTACTCATTATCTGAGGAGCCGGTCAAATATTCA	INDEL	51	Gain-of-function	18.0	2	p.Phe594_Arg595insSerGluTyrLeuThrGlySerSerAspAsnGluTyrPheTyrValAspPhe	932	14	c.1784_1785insTGAATATTTGACCGGCTCCTCAGATAATGAGTACTTCTACGTTGATTTCAG	nonframeshiftInsertion
	FLT3	chr13:28608276	A/GGAGCCGGTCAAATATTCACTGAA	A	GGAGCCGGTCAAATATTCACTGAA	FLT3ITD	24	Gain-of-function	27.6	2	p.Arg595fs	497	14	c.1780_1780delTinsTTCAGTGAATATTTGACCGGCTCC	frameshiftBlockSubstitution

Table S11-1. Kasumi-2

Genes	Locus	Ref	Type	Kasumi-2	Variant Frequency	Location	Amino Acid Change	Coding
RUNX3	chr1:25228945	T	SNV	T/C	15.0	RUNX3:exonic:NM_004350.2	p.Thr306Ala	c.916A>G
TAL1	chr1:47691544	G	SNV	G/A	46.2	TAL1:exonic:NM_003189.5	p.Pro6Leu	c.17C>T
TET2,	chr4:106156139	C	SNV	C/T	54.1	TET2:exonic:NM_001127208.2	p.Ala347Val	c.1040C>T
NSD1	chr5:176638269	G	SNV	G/A	21.4	NSD1:exonic:NM_022455.4	p.Val957Ile	c.2869G>A
CARD11	chr7:2987368	C	SNV	T/T	100.0	CARD11:exonic:NM_032415.5	p.Ala21Thr	c.61G>A
HGF	chr7:81350061	C	SNV	C/T	19.9	HGF:exonic:NM_000601.5	p.Arg424His	c.1271G>A
HGF	chr7:81350089	A	SNV	A/G	20.8	HGF:exonic:NM_000601.5	p.Trp415Arg	c.1243T>C
RET	chr10:43604485	T	SNV	T/C	11.0	RET:exonic:NM_020975.4	p.Val357Ala	c.1070T>C
PTEN	chr10:89711903	A	SNV	A/G	11.5	PTEN:exonic:NM_000314.6	p.Tyr174Cys	c.521A>G
KRAS	chr12:25398279	C	SNV	C/G	48.4	KRAS:exonic:NM_033360.3	p.Val14Leu	c.40G>C
TP53	chr17:7579882	C	SNV	C/G	44.8	TP53:exonic:NM_000546.5	p.Glu11Gln	c.31G>C
NF1	chr17:29653162	G	SNV	G/T	55.4	NF1:exonic:NM_001042492.2	p.Glu1720Asp	c.5160G>T
PRKAR1A	chr17:66524000	G	SNV	G/A	10.1	PRKAR1A:exonic:NM_212471.2	p.Arg243Gln	c.728G>A
HCK	chr20:30659499	G	SNV	G/A	47.4	HCK:exonic:NM_001172133.1	p.Gly12Arg	c.34G>A
VSIG4	chrX:65252402	G	SNV	G/A	76.9	VSIG4:exonic:NM_007268.2	p.Ala201Val	c.602C>T
ATRX	chrX:76940462	G	SNV	A/A	100.0	ATRX:exonic:NM_000489.4	p.Arg211Cys	c.631C>T

Table S11-2. Kasumi-5

Genes	Locus	Ref	Type	Kasumi-5	Variant Frequency	Location	Amino Acid Change	Coding
FIP1L1	chr4:54319259	GAG	INDEL	GAG/G	11.8	FIP1L1:exonic:NM_001134937.1	p.Arg481fs	c.1441_1442delAG
KIT	chr4:55593461	G	SNV	G/C	57.1	KIT:exonic:NM_000222.2	p.Val540Leu	c.1618G>C
KDR	chr4:55980324	A	SNV	A/G	51.2	KDR:exonic:NM_002253.2	p.Ile256Thr	c.767T>C
IL7R	chr5:35874563	C	SNV	C/G	39.7	IL7R:exonic:NM_002185.4	p.Pro240Arg	c.719C>G
IL7R	chr5:35874565	A	SNV	A/G	39.7	IL7R:exonic:NM_002185.4	p.Ile241Val	c.721A>G
IL7R	chr5:35874569	TA	INDEL	TA/T	39.7	IL7R:exonic:NM_002185.4	p.Leu242fs	c.726delA
NSD1	chr5:176638269	G	SNV	G/A	23.5	NSD1:exonic:NM_022455.4	p.Val957Ile	c.2869G>A
NSD1	chr5:176696787	G	SNV	G/C	59.7	NSD1:exonic:NM_022455.4	p.Val1830Leu	c.5488G>C
NOTCH1	chr9:139399368	A	SNV	A/G	46.7	NOTCH1:exonic:NM_017617.4	p.Phe1592Ser	c.4775T>C
SVIL	chr10:29840012	C	SNV	C/T	41.5	SVIL:exonic:NM_021738.2	p.Arg114Gln	c.341G>A
HNF1A	chr12:121416797	G	SNV	G/A	49.3	HNF1A:exonic:NM_000545.6	p.Asp76Asn	c.226G>A
CREBBP	chr16:3778300	T	SNV	T/C	51.5	CREBBP:exonic:NM_004380.2	p.Met2250Val	c.6748A>G
TP53	chr17:7579350	A	SNV	T/T	98.6	TP53:exonic:NM_000546.5	p.Phe113Ile	c.337T>A
SETBP1	chr18:42533093	A	SNV	A/G	56.7	SETBP1:exonic:NM_015559.2	p.Tyr1263Cys	c.3788A>G

Table S11-3. Kasumi-7

Genes	Locus	Ref	Type	Kasumi-7	Variant Frequency	Location	Amino Acid Change	Coding
TRH	chr3:129695847	G	SNV	G/A	51.8	TRH:exonic:NM_007117.4	p.Glu173Lys	c.517G>A
TRIO	chr5:14381290	T	SNV	T/G	63.6	TRIO:exonic:NM_007118.3	p.Ser1167Ala	c.3499T>G
PDGFRB	chr5:149502735	G	SNV	G/A	48.2	PDGFRB:exonic:NM_002609.3	p.Arg685Cys	c.2053C>T
NSD1	chr5:176638269	G	SNV	G/A	25.9	NSD1:exonic:NM_022455.4	p.Val957Ile	c.2869G>A
NSD1	chr5:176638327	C	SNV	C/T	53.1	NSD1:exonic:NM_022455.4	p.Ala976Val	c.2927C>T
ABL1	chr9:133730221	A	SNV	A/G	43.9	ABL1:exonic:NM_005157.5	p.Asn96Ser	c.287A>G
ABL1	chr9:133760303	A	SNV	A/G	59.4	ABL1:exonic:NM_005157.5	p.Arg876Gly	c.2626A>G
PYGL	chr14:51390767	G	SNV	G/A	51.8	PYGL:exonic:NM_002863.4	p.Arg194Cys	c.580C>T
TOP2A	chr17:38572272	C	SNV	C/T	47.3	TOP2A:exonic:NM_001067.3	p.Arg106Lys	c.317G>A

Table S11-4. Kasumi-8

Genes	Locus	Ref	Type	Kasumi-8	Variant Frequency	Location	Amino Acid Change	Coding
APC	chr5:112177427	G	SNV	G/T	50.6	APC:exonic:NM_000038.5	p.Ala2046Ser	c.6136G>T
NSD1	chr5:176638269	G	SNV	G/A	26.4	NSD1:exonic:NM_022455.4	p.Val957Ile	c.2869G>A
ETV6	chr12:12006443	A	INDEL	AAG/AAG	99.0	ETV6:exonic:NM_001987.4	p.Asn138fs	c.412_413insGA

Table S11-5. Kasumi-9

Genes	Locus	Ref	Type	Kasumi-9	Variant Frequency	Location	Amino Acid Change	Coding
RPL5	chr1:93299200	A	SNV	A/G	17.2	RPL5:exonic:NM_000969.4	p.Arg58Gly	c.172A>G
RPL5	chr1:93299205	TATCAT	INDEL	TATCAT/T	17.3	RPL5:exonic:NM_000969.4	p.Ile60fs	c.178_182delATCAT
RPL5	chr1:93303115	C	INDEL	C/CT	31.6	RPL5:exonic:NM_000969.4	p.Leu211fs	c.632_633insT
GLI2	chr2:121748219	G	SNV	G/A	47.9	GLI2:exonic:NM_005270.4	p.Glu1577Lys	c.4729G>A
TRH	chr3:129695847	G	SNV	G/A	50.8	TRH:exonic:NM_007117.4	p.Glu173Lys	c.517G>A
MECOM	chr3:168834110	C	SNV	C/T	31.5	MECOM:exonic:NM_004991.3	p.Ser517Asn	c.1550G>A
NSD1	chr5:176638269	G	SNV	G/A	26.3	NSD1:exonic:NM_022455.4	p.Val957Ile	c.2869G>A
DDX41	chr5:176938914	C	SNV	C/T	18.9	DDX41:exonic:NM_016222.3	p.Ala583Thr	c.1747G>A
BMP6	chr6:7862663	G	SNV	G/A	52.2	BMP6:exonic:NM_001718.5	p.Arg379Gln	c.1136G>A
EZH2	chr7:148526829	C	SNV	C/T	18.6	EZH2:exonic:NM_004456.4	p.Gly159Arg	c.475G>A
JAK2	chr9:5080268	T	SNV	T/C	54.1	JAK2:exonic:NM_004972.3	p.Ile724Thr	c.2171T>C
SPI1	chr11:47377073	T	SNV	T/A	47.2	SPI1:exonic:NM_001080547.1	p.Tyr174Phe	c.521A>T
NCOA3	chr20:46279851	GCAGCAGCAGCAGCAA	INDEL	GCAGCAGCAGCAGCAA/G	33.3	NCOA3:exonic:NM_181659.2	p.Gln1260_Gln1264del	c.3783_3797delGCAGCAGCAACAGCA

Table S11-6. Kasumi-10

Genes	Locus	Ref	Type	Kasumi-10	Variant Frequency	Location	Amino Acid Change	Coding
NSD1	chr5:176638269	G	SNV	G/A	18.5	NSD1:exonic:NM_022455.4	p.Val957Ile	c.2869G>A
C6orf10	chr6:32260907	C	INDEL	C/CT	16.9	C6orf10:exonic:NM_006781.4	p.Glu515fs	c.1542_1543insA
GATA3	chr10:8115837	G	SNV	G/A	47.1	GATA3:exonic:NM_001002295.1	p.Ala396Thr	c.1186G>A
FLT3	chr13:28608270	C	INDEL	C/CAGGGAAATCTTCACTGAAATCAACGTAGAAGTACTCATTA	35.9	FLT3:exonic:NM_004119.2	p.Glu596Ter	c.1785_1786insTAATGAGTACTTCTACGTTGATTTCAGTGAAGATTTCCCT
FLT3	chr13:28608275	A	INDEL	A/AGGAGCCGGTCAAATATTCACTGA	34.0	FLT3:exonic:NM_004119.2	p.Arg595fs	c.1780_1781insTCAGTGAATATTTGACCGGCTCC
FLT3	chr13:28623544	T	SNV	T/G	48.9	FLT3:exonic:NM_004119.2	p.Gln338Pro	c.1013A>C
PER1	chr17:8053928	G	SNV	G/A	49.3	PER1:exonic:NM_002616.2	p.His33Tyr	c.97C>T

Table S12. Fusion genes identified by the Oncomine Myeloid panel

Sample	Genes (Exons)	Read Counts	Read Counts Per Million	Variant ID	Locus	Translocation
Kasumi-1	RUNX1(3) - RUNX1T1(3)	5748	117471.5	RUNX1-RUNX1T1.R3R3	chr21:36231771 - chr8:93029591	t(8;21)(q21.3;q22.1)
Kasumi-2	TCF3(16) - PBX1(3)	2967	231074.8	TCF3-PBX1.T16P3.COSF1489	chr19:1619110 - chr1:164761731	t(1;19)(q23.3;p13.3)
Kasumi-4	BCR(13) - ABL1(2)	1073	34680.0	BCR-ABL1.B13A2	chr22:23631808 - chr9:133729451	t(9;22;11)(q34.1;q11.2;q13)
Kasumi-8	BCR(1) - ABL1(2)	1067	46589.8	BCR-ABL1.B1A2	chr22:23524426 - chr9:133729451	t(9;22)(q34.1;q11.2)
Kasumi-10	KMT2A(9) - MLLT1(2)	200	9559.8	KMT2A-MLLT1.K9M2	chr11:118355029 - chr19:6270770	t(11;19)(q23.3;p13.3)
	KMT2A(10) - MLLT1(2)	232	11089.3	KMT2A-MLLT1.K10M2	chr11:118355690 - chr19:6270770	t(11;19)(q23.3;p13.3)

Table S14. Comparison of cell marker expression from flow cytometric analysis

		CD3		CD4		CD8		CD10		CD13		CD14		CD16		CD19		CD20		CD22	
Kasumi-3	AML	-	0.1	++	59.4	-	0.3	+-	3.1	+	37.0	-	0.3	-	0.9	-	0	-	0.1	+	36.3
Kasumi-1	AML	-	0.2	+-	14.9	-	1.5	-	1.5	-	1.9	-	0.9	-	1.2	-	0.2	-	0.3	-	0.7
Kasumi-6	AML	-	0.1	-	0.9	-	0.1	-	0.2	+-	2.1	-	0.1	-	0.2	-	0.1	-	0	-	0.1
Kasumi-4	CML	-	0.0	+	39.1	-	0.2	-	0.5	+	37.7	-	0.2	-	0.5	-	0.1	-	0	-	0.6
Kasumi-5	T-ALL	-	0.7	-	0.8	-	0.3	++	67.4	+-	4.1	-	1.6	-	0.8	-	0	-	0.3	-	0.6
Kasumi-2	BCP-ALL	-	0.1	-	0.0	-	0.4	++	72.8	-	1.8	-	0.1	-	0.4	+++	97.6	+	27.1	+++	97.5
Kasumi-7	BCP-ALL	-	0.2	-	0.3	-	0	-	0.8	-	0.2	-	0.7	-	0.1	++	54.1	-	0.3	++	65.3
Kasumi-8	BCP-ALL	-	0.2	+-	2.8	-	0.1	+++	100	-	0.3	-	0.4	-	0.3	+++	99.8	-	0.2	+++	100
Kasumi-9	BCP-ALL	-	0.2	-	0.4	-	0.8	+-	2.4	-	0.1	-	0.4	-	1.1	+++	99.1	+-	5.6	+++	99.7
Kasumi-10	BCP-ALL	-	0.3	-	0.7	-	0.5	-	1.1	-	1.4	-	2.0	-	0.6	+++	85.8	-	0.5	+	44.4

		CD23		CD25		CD30		CD33		CD34		CD38		CD45		CD56		HLA-A,B,C		HLA-DR	
Kasumi-3	AML	-	0.1	-	0.1	-	0.1	+	45.3	+++	100	+++	97.0	+++	99.3	+++	80.6	+++	95.4	+-	7.6
Kasumi-1	AML	-	0.5	-	0.2	-	0.6	+-	13.7	+	41.5	+-	14.3	++	53.4	-	0.6	+++	93.3	-	0.9
Kasumi-6	AML	-	0	-	0.9	-	0.2	++	63.7	-	0.2	+	39.3	+	36.5	-	0.1	+++	99.2	-	0.3
Kasumi-4	CML	-	0	+-	4.2	-	0.5	+-	14.9	+-	5.6	-	1.2	+++	99.1	+++	92.5	+++	100	+-	13.1
Kasumi-5	T-ALL	-	0.5	-	0.2	+-	16.7	-	1.1	-	0.4	+++	100	+++	85.9	+-	12.0	+++	99.9	-	0.2
Kasumi-2	BCP-ALL	-	0.1	-	0.1	+-	3.2	-	0.1	-	0.6	+++	99.2	+++	91.7	-	0.1	+++	100	+-	5.4
Kasumi-7	BCP-ALL	-	0.2	-	0.3	-	0.3	-	0.9	-	0.2	+++	96.7	-	0.3	-	0.2	+++	100	+-	2.1
Kasumi-8	BCP-ALL	-	0.3	-	0.1	-	0.1	-	0.2	-	0.1	+++	96.3	++	64.1	-	0.2	+++	100	++	54.6
Kasumi-9	BCP-ALL	-	0.3	-	0.1	+-	12.4	-	0.3	-	1.2	+++	99.8	-	0.2	-	0.4	+++	100	++	64.8
Kasumi-10	BCP-ALL	+-	9.9	+-	2.9	-	0.3	+-	4.8	-	0.5	+++	98.6	+++	89.3	-	0.4	+++	99.9	+	47.7
